# Integrative omics analysis incorporating cardiovascular magnetic resonance imaging pinpoints potentially druggable plasma proteins for cardiovascular diseases

**DOI:** 10.1093/lifemeta/loag001

**Published:** 2026-01-07

**Authors:** Weiming Gong, Ping Guo, Lu Liu, Xiubin Sun, Shukang Wang, Fuzhong Xue, Lujia Shen, Zhongshang Yuan

**Affiliations:** Department of Biostatistics, School of Public Health, Cheeloo College of Medicine, Shandong University, Jinan, Shandong 250012, China; Institute for Medical Dataology, Shandong University, Jinan, Shandong 250003, China; Department of Biostatistics, School of Public Health, Cheeloo College of Medicine, Shandong University, Jinan, Shandong 250012, China; Institute for Medical Dataology, Shandong University, Jinan, Shandong 250003, China; Department of Biostatistics, University of Michigan, Ann Arbor, MI 48109, United States; Center for Statistical Genetics, University of Michigan, Ann Arbor, MI 48109, United States; Department of Biostatistics, School of Public Health, Cheeloo College of Medicine, Shandong University, Jinan, Shandong 250012, China; Institute for Medical Dataology, Shandong University, Jinan, Shandong 250003, China; Department of Biostatistics, School of Public Health, Cheeloo College of Medicine, Shandong University, Jinan, Shandong 250012, China; Institute for Medical Dataology, Shandong University, Jinan, Shandong 250003, China; Department of Biostatistics, School of Public Health, Cheeloo College of Medicine, Shandong University, Jinan, Shandong 250012, China; Institute for Medical Dataology, Shandong University, Jinan, Shandong 250003, China; The Second Affiliated Hospital, School of Public Health, Zhejiang Key Laboratory of Intelligent Preventive Medicine, School of Medicine, Zhejiang University, Hangzhou, Zhejiang 310058, China; Department of Biostatistics, School of Public Health, Cheeloo College of Medicine, Shandong University, Jinan, Shandong 250012, China; Institute for Medical Dataology, Shandong University, Jinan, Shandong 250003, China

**Keywords:** cardiovascular disease, cardiovascular magnetic resonance imaging, druggable plasma proteins, proteome-wide association study, Mendelian randomization, colocalization

## Abstract

Despite advances in traditional risk factors for cardiovascular diseases (CVDs), significant residual risk of CVDs remains incompletely captured. Integrative analysis incorporating cardiovascular magnetic resonance imaging (CMR) could facilitate to discover novel ­therapeutic targets. This study aimed to identify potentially druggable plasma proteins for CVDs by incorporating CMR traits with integrative omics analysis. By integrating protein quantitative trait loci (pQTL) datasets of plasma proteins from Atherosclerosis Risk in Communities (ARIC) study with genome-wide association studies of 19 CVDs and 82 CMR traits, we sequentially used proteome-wide association study (PWAS), Mendelian randomization (MR), and colocalization analysis to identify putatively causal proteins. Replication MR was conducted using deCODE pQTL data, followed by observational association analysis using UK Biobank individual-level data, and multidimensional downstream analyses, as well as phenome-wide MR (Phe-MR). In total, we identified 342 protein–CVD and 115 protein–CMR pairs through PWAS. MR and colocalization ana­lyses revealed 66 protein–CVD and 39 protein–CMR pairs with potential causal relationships, of which 51 protein–CVD and 33 protein–CMR pairs were replicated. Additionally, 26 protein–CVD and 6 protein–CMR pairs showed significantly observational associations. Multidimensional downstream analysis highlighted potential biological pathways and druggability insights. Notably, *AGER*, *CCN3*, *FER*, and *SPON1* were identified as proteins associated with both CVDs and CMR traits. Phe-MR analysis suggests potential beneficial and adverse effects of these proteins on other diseases. Our findings highlight potentially druggable plasma proteins for CVDs by incorporating CMR traits, providing novel insights into CVD pathogenesis and therapeutic drug development.

## Introduction

Cardiovascular diseases (CVDs), comprising a spectrum of cardiac and vascular diseases, were well documented to be the leading cause of morbidity, disability, and premature death worldwide [1, 2]. Currently, employing favorable lifestyles to reduce cardiovascular risk factors, in conjunction with pharmacotherapy, is a primary regime for CVD management [3]. However, even under optimal treatment conditions, there remains significant residual risk that is incompletely captured by traditional risk factors and existing therapies [4]. The discovery of novel therapeutic targets or the repurposing of existing drug targets could improve the prevention and treatment for different CVDs [5].

Plasma proteome mirrors the physiological state of the cardiovascular system [6] and could serve as potential druggable targets [7]. Emerging genome-wide association studies (GWAS) of CVD and protein quantitative trait loci (pQTL) studies, together with statistical genetics methods developed to integrate pQTL and GWAS data, provide great opportunities to facilitate the discovery of protein–disease associations. In particular, proteome-wide association study (PWAS) aims to identify potential disease-related proteins [8]. Mendelian randomization (MR) could explore the causal effect of proteins on complex traits, which is less susceptible to confounding bias and reverse causation [9]. Colocalization analysis could examine the shared causal variants between proteins and diseases [10]. Previous studies have implemented some of these techniques to explore the associations between plasma proteins and cardiovascular outcomes [11, 12]. However, these studies encounter the following analytic challenges.

First, these studies fail to cover the whole spectrum of CVDs and lack comprehensive analysis framework. Indeed, using different methods could complement to each other and improve reliability of findings. For example, PWAS could be adopted before MR to initially select the proteins associated with CVDs, which could reduce the burden of multiple testing in the subsequent MR ana­lysis [13]. Second, most of these studies have not included cardiovascular magnetic resonance imaging (CMR) traits into analysis. CMR is an important tool to quantify and assess cardiovascular structure and function, and often used for the diagnosis of CVDs [14, 15]. For instance, the left ventricular ejection fraction is an important ­clinical biomarker for the diagnosis and treatment of heart failure (HF) [16]; similarly, the left ventricular myocardial mass is used to classify hypertrophy and predict the risk of cardiovascular events [17]. Incorporating multiple CMR traits in the ana­lysis could help to disentangle the complex relationships among proteins, CMR traits, and CVDs, which may provide further insights into the ­discovery of potential drug targets. Currently, there are only two protein–CVD association studies with the CMR traits included, but both are restricted to a small number of CMR traits [11, 12]. Third, these studies lack large-scale replication analysis and multidimensional downstream analyses, leading to less reproducible results and insufficient biological explanations.

**Figure 1:**
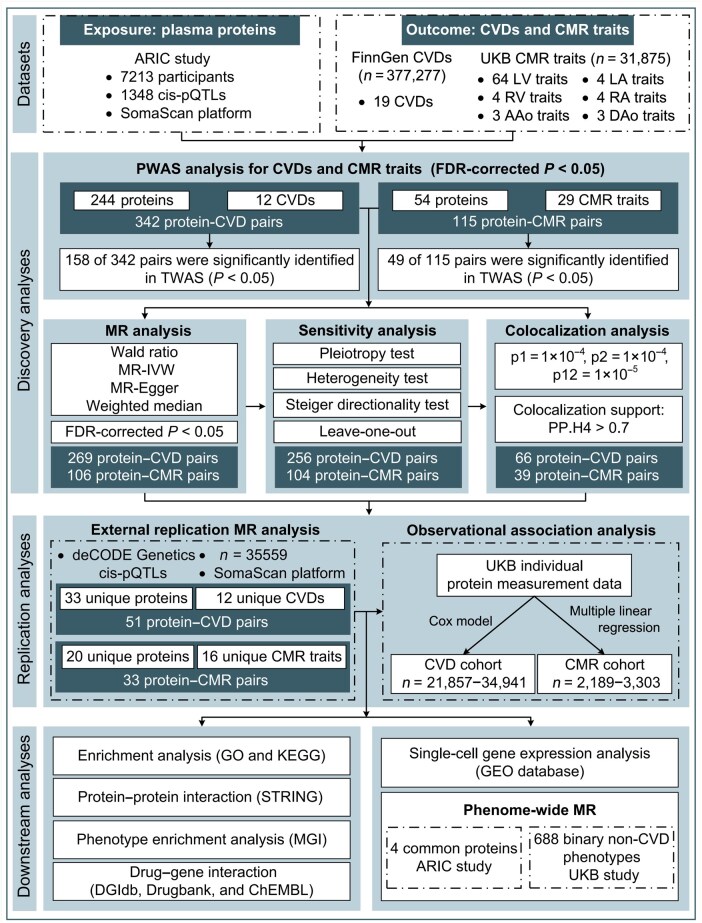
The flowchart of the study design. We conducted a systematical large-scale omics-integration analysis by sequentially performing discovery, replication, and multidimensional downstream analysis incorporating CMR traits to investigate potentially druggable plasma proteins for CVDs. ARIC, Atherosclerosis Risk in Communities; pQTL, protein quantitative trait loci; CVD, cardiovascular disease; CMR, cardiovascular magnetic resonance imaging; UKB, UK Biobank; LV, left ventricle; LA, left atrium; RV, right ventricle; RA, right atrium; AAo, ascending aorta; DAo, descending aorta; PWAS, proteome‑wide association study; FDR, false discovery rate; TWAS, transcriptome-wide association study; MR, Mendelian randomization; IVW, inverse-variance weighted; PP.H4, posterior probability of H4; GO, Gene Ontology; KEGG, Kyoto Encyclopedia of Genes and Genomes; MGI, Mouse Genome Informatics platform; DGIdb, the Drug-Gene Interaction Database; GEO, Gene Expression Omnibus.

In this study, we aimed to identify proteins associated with 19 CVDs and 82 CMR traits by a sequential analysis pipeline with various statistical genetic approaches incorporated. In discovery analysis, we implemented parallel PWAS analyses to identify protein–CVD and protein–CMR associations, followed by two-sample MR and colocalization analyses to screen potentially causal proteins. In replication analysis, we conducted MR analysis using an independent pQTL data and conducted observational association analysis using UK Biobank (UKB) individual level data. Furthermore, we integrated multidimensional downstream ­analyses to investigate the underlying biological mechanisms and provide genetic evidence to pinpoint potentially druggable proteins for CVDs. An overview of the study design is displayed in Fig. 1.

## Results

### Discovery of potentially causal protein–CVD and protein–CMR associations

In discovery analysis, we identified 342 significant protein–CVD pairs involving 244 unique protein-coding genes associated with at least 1 of 12 CVDs (Supplementary Table S1) and 115 significant protein–CMR pairs involving 54 unique genes associated with at least 1 of 29 CMR traits through PWAS (Supplementary Table S2). Notably, 23 unique protein-coding genes were found to be associated with both CVD and CMR trait. For example, advanced glycosylation end-product specific receptor (*AGER*) was suggested to be not only negatively associated with coronary atherosclerosis (CAS; *P *= 2.94 × 10^−5^), hypertension (HYPTE; *P *= 1.51 × 10^−4^), and myocardial infarction (MI; *P *= 3.65 × 10^−8^) but also with ascending aorta maximum area (AAo_max_area; *P *= 4.58 × 10^−4^), ascending aorta minimum area (AAo_min_area; *P *= 2.25 × 10^−4^), and left ventricular cardiac output (LVCO; *P *= 9.98 × 10^−5^). In addition, 46.2% (158 of 342) protein–CVD associations (Supplementary Fig. S1; Supplementary Tables S1 and S3) and 42.6% (49 of 115) protein–CMR associations (Supplementary Fig. S2; Supplementary Tables S2 and S4) were further replicated by transcriptome-wide association studies (TWAS).

Through primary MR analysis together with various sensitivity analyses, a total of 256 potentially causal protein–CVD pairs were identified (Supplementary Fig. S3; Supplementary Tables S5–S7), involving 182 unique proteins associated with at least 1 of 12 CVDs, which showed consistent effect directions with PWAS findings, with 140 pairs showing positive associations and 116 pairs showing ­negative associations. Totally, 104 potentially causal protein–CMR pairs were detected (Supplementary Fig. S4; Supplementary Tables S8–S10), involving 51 unique proteins associated with at least 1 of 27 CMR traits, which, again, were consistent with the PWAS results in terms of the effect directions, with 50 pairs showing positive associations and 54 pairs showing negative associations. As sensitivity analysis of primary MR analysis, we retained all palindromic single nucleotide polymorphisms (SNPs) to perform sensitivity MR analysis, and found strong correlations and consistent directions between the causal estimates from the sensitivity MR analysis and primary MR analysis (278 versus 269 protein–CVD pairs, 268 were overlapped: *r *= 0.997, *P *< 2.2 × 10^−16^; 106 versus 106 protein–CMR pairs, 106 were overlapped: *r *= 1.000, *P *< 2.2 × 10^−16^; Supplementary Tables S11 and S12). Further colocalization analysis identified 25.8% (66 of 256) protein–CVD pairs and 37.5% (39 of 104) protein–CMR pairs with the posterior probability of hypothesis 4 (PP.H4) larger than 0.7 (Supplementary Tables S13 and S14).

Notably, five proteins showed causal associations with both CVD and CMR trait and were robust against potential outlier ­instruments (Supplementary Fig. S5), including the effect of *AGER* on LVCO and MI, cellular communication network factor 3 (*CCN3*) on left ventricular end-systolic volume (LVESV) and aortic aneurysm (AA), FER tyrosine kinase (*FER*) level on descending aorta maximum area (DAo_max_area), descending aorta minimum area (DAo_min_area), AA and varicose veins (VV), haptoglobin (*HP*) on AAo_min_area and atherosclerosis (excluding cerebral, coronary, and PAD) (PAS), and spondin 1 (*SPON1*) on global longitudinal strain (Ecc_global), LVESV, and atrial fibrillation and flutter (AF).

### External replication and observational evidence

**Figure 2:**
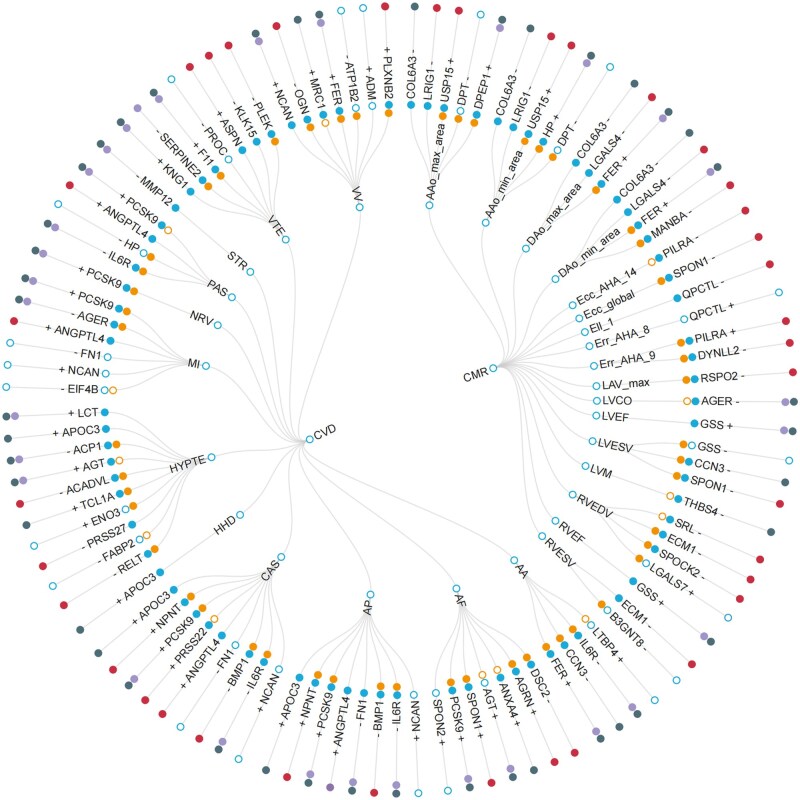
Overall landscape of potentially druggable proteins influencing CVDs and CMR traits. A circular dendrogram involved 12 CVDs and 17 CMR traits (second circle) are shown. A total of 66 protein–CVD associations and 39 protein–CMR associations were identified by sequential PWAS, MR, and colocalization approaches in discovery analysis (2 associations of *KNG1*-VTE, *PILRA*-Ecc_AHA_14, and *PILRA*-Err_AHA_9 were displayed once). TWAS partly replicated the results at transcript level (third circle; orange dots, TWAS-significant signal; orange circles, TWAS non-significant signal). The protein–CVD pairs (51 of 66) and protein–CMR pairs (33 of 39) were significantly replicated with the effect directions consistent with that in discovery analysis (fourth circle; bright cyan dots, replicated; bright cyan circle, not replicated), with the effect directions marked (protective effect on CVD and decrease CMR measurement were marked by –, while risk effect on CVD and increase CMR measurement were marked by +). We prioritized the potentially druggable targets by classifying these protein-coding genes into three categories (outer circle; dark blue dots, approved [already served as therapeutic targets]; lavender gray dots, druggable [reported in clinical trials]; bright maroon dots, untargeted [no current druggable evidence]). CVD, cardiovascular diseases; CMR, cardiovascular magnetic resonance; AA, aortic aneurysm; AF, atrial fibrillation and flutter; AP, angina pectoris; CAS, coronary atherosclerosis; HHD, hypertensive heart disease; HYPTE, hypertension; MI, myocardial infarction; NRV, rheumatic valve diseases; PAS, atherosclerosis, excluding cerebral, coronary and PAD; STR, stroke; VTE, venous thromboembolism; VV, varicose veins. AAo_max_area, ascending aorta maximum area; AAo_min_area, ascending aorta minimum area; DAo_max_area, descending aorta maximum area; DAo_min_area, descending aorta minimum area; Ecc_AHA_14, regional peak circumferential strain; Ecc_global, global peak circumferential strain; Ell_1, regional longitudinal strain; Err_AHA_8, regional radial strain; Err_AHA_9, regional radial strain; LAV_max, left atrium maximum volume; LVCO, left ventricular cardiac output; LVEF, left ventricular ejection fraction; LVESV, left ventricular end-systolic volume; LVM, left ventricular myocardial mass; RVEDV, right ventricular end-diastolic volume; RVEF, right ventricular ejection fraction; RVESV, right ventricular end-systolic volume.

**Figure 3:**
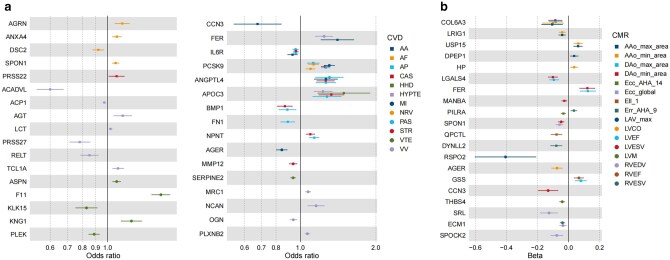
Causal associations of protein–CVDs or protein–CMR traits in external replication analysis. (a) The causal effects of proteins on CVDs in external MR replication analysis. (b) The causal effects of proteins on CMR traits in external MR replication analysis. AA, aortic aneurysm; AF, atrial fibrillation and flutter; AP, angina pectoris; CAS, coronary atherosclerosis; HHD, hypertensive heart disease; HYPTE, hypertension; MI, myocardial infarction; NRV, rheumatic valve diseases; PAS, atherosclerosis, excluding cerebral, coronary and PAD; STR, stroke; VTE, venous thromboembolism; VV, varicose veins. AAo_max_area, ascending aorta maximum area; AAo_min_area, ascending aorta minimum area; DAo_max_area, descending aorta maximum area; DAo_min_area, descending aorta minimum area; Ecc_AHA_14, regional peak circumferential strain; Ecc_global, global peak circumferential strain; Ell_1, regional longitudinal strain; Err_AHA_9, regional radial strain; LAV_max, left atrium maximum volume; LVCO, left ventricular cardiac output; LVEF, left ventricular ejection fraction; LVESV, left ventricular end-systolic volume; LVM, left ventricular myocardial mass; RVEDV, right ventricular end-diastolic volume; RVEF, right ventricular ejection fraction; RVESV, right ventricular end-systolic volume.

**Figure 4:**
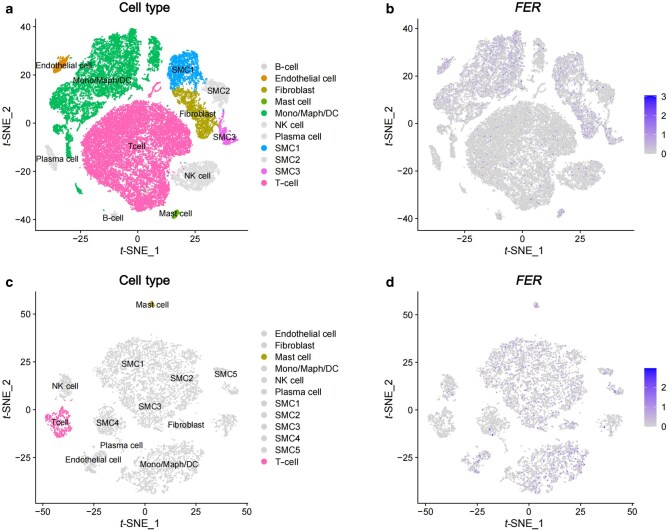
Single-cell expression of *FER* gene in ascending thoracic AA cases and controls, respectively. (a) A total of 11 cell types identified in eight cases. Cell types with differential expression of *FER* gene are annotated in color. (b) Expression of *FER* gene in each cell type of eight cases. (c) A total of 12 cell types identified in three controls. Cell types with differential expression of *FER* gene are annotated in color. (d) Expression of *FER* gene in each cell type of three controls.

In replication MR analysis, 51 of 66 protein–CVD pairs (involving 33 unique proteins and 12 CVDs) and 33 of 39 protein–CMR pairs (involving 20 unique proteins and 16 unique CMR traits) were ­replicated with the effect directions consistent with that in discovery analysis (Figs. 2 and 3), 4 of the 5 common proteins (AGER, CCN3, FER, and SPON1) passed the replication analysis (Table 1), while *HP*-PAS showing broader evidence (false discovery rate [FDR]-corrected *P *= 0.06) (Supplementary Fig. S6; Supplementary Tables S15–S18). As sensitivity analysis of replication MR analysis, we performed sensitivity MR analysis again by retaining all palindromic SNPs, and we also found strong and consistent correlations between the causal estimates from the sensitivity MR analysis and replication MR analysis (51 versus 51 protein–CMR pairs, 50 were overlapped: *r *= 0.999, *P *< 2.2 × 10^−16^; 34 versus 33 protein–CMR pairs, 33 were overlapped: *r *= 1.000, *P *< 2.2 × 10^−16^; Supplementary Tables S19 and S20).

**Table 1:** Four common proteins associated with both CVDs and CMR traits.

Gene	CMR trait	Discovery				Replication	CVD	Discovery				Replication
		**PWAS.Z (*P*** ^a^ **)**	TWAS Significant	**MR.Beta (*P*** ^a^ **)**	Coloc PP.H4	**MR.Beta (*P*** ^a^ **)**		**PWAS.Z (*P*** ^a^ **)**	TWAS Significant	**MR.OR (*P*** ^a^ **)**	Coloc PP.H4	**MR.OR (*P*** ^a^ **)**
***AGER***	LVCO	−3.891 (4.44 × 10^−2^)	No	−0.075 (2.18 × 10^−4^)	0.923	−0.077 (2.25 × 10^−4^)	MI	−5.507 (2.25 × 10^−4^)	Yes	0.843 (5.57 × 10^−9^)	0.995	0.914 (2.18 × 10^−2^)
***CCN3***	LVESV	−3.908 (2.72 × 10^−2^)	Yes	−0.132 (2.18 × 10^−4^)	0.872	−0.390 (1.11 × 10^−4^)	AA	−4.367 (5.43 × 10^−3^)	Yes	0.676 (1.58 × 10^−3^)	0.726	0.351 (8.44 × 10^−4^)
***FER***	DAo_max_area	5.104 (4.45 × 10^−4^)	Yes	0.123 (3.96 × 10^−5^)	0.965	0.264 (8.10 × 10^−4^)	AA	4.309 (5.43 × 10^−3^)	Yes	1.405 (1.21 × 10^−4^)	0.935	1.999 (2.21 × 10^−3^)
	**DAo_min_area**	5.072 (4.45 × 10^−4^)	Yes	0.120 (4.32 × 10^−5^)	0.959	0.257 (8.97 × 10^−4^)	VV	5.118 (5.85 × 10^−5^)	Yes	1.243 (2.64 × 10^−6^)	0.966	1.490 (9.97 × 10^−4^)
***SPON1***	Ecc_global	−4.619 (5.14 × 10^−3^)	Yes	−0.059 (3.04 × 10^−5^)	0.983	−0.052 (4.42 × 10^−2^)	AF	4.546 (1.45 × 10^−3^)	Yes	1.075 (1.21 × 10^−4^)	0.835	1.100 (1.10 × 10^−2^)
	**LVESV**	−4.431 (5.14 × 10^−3^)	Yes	−0.048 (4.95 × 10^−5^)	0.959	−0.052 (5.62 × 10^−3^)						

a False discovery rate (FDR)-corrected *P* values. CVD, cardiovascular diseases; CMR, cardiovascular magnetic resonance; PWAS, proteome-wide association study; TWAS, transcriptome-wide association study; MR, Mendelian randomization; Coloc, colocalization; PP.H4, posterior probability of H4; OR, odds ratio; LVCO, left ventricular cardiac output; LVESV, left ventricular end-systolic volume; DAo_max_area, descending aorta maximum area; DAo_min_area, descending aorta minimum area; Ecc_global, global peak circumferential strain; MI, myocardial infarction; AA, aortic aneurysm; VV, varicose veins; AF, atrial fibrillation and flutter.

Observational association analysis was performed on protein–CVD and protein–CMR associations passed external replication. Due to the absence of 8 proteins (including *FER*), 36 among 51 protein–CVD pairs were analyzed, 26 of 36 pairs (72%) showed significant associations (Supplementary Table S21), including *CCN3*−AA and *SPON1*−AF, in which seven pairs had the opposite effect directions with that in discovery analysis, which was possibly due to small sample size, ­population heterogeneity, or potential confounding bias. No significant association was found between *AGER* and MI (FDR-corrected *P *= 0.620). Due to the absence of 10 proteins, 20 protein–CMR pairs were analyzed, and 6 of 20 (30%) pairs showed significant associations with consistent effect directions with that in discovery analysis (Supplementary Table S22), including *AGER*−LVCO and *SPON1*−LVESV. Nominally significant association was found between *SPON1* and Ecc_global (*P *= 0.034).

### Characterization of biological mechanisms and prioritization of druggable proteins

For 33 CVD-associated protein-coding genes passed external replication analysis, 79 Gene Ontology (GO) terms and 4 Kyoto Encyclopedia of Genes and Genomes (KEGG) pathways were identified (Supplementary Fig. S7a; Supplementary Table S23), such as smooth muscle cell proliferation (*P *= 1.86 × 10^−4^), negative regulation of low-density lipoprotein particle clearance (*P *= 2.55 × 10^−4^), and cholesterol metabolism (*P *= 2.24 × 10^−4^). For 20 CMR-associated protein-coding genes passed external replication analysis, enrichment analysis identified 17 GO terms (Supplementary Fig. S7b; Supplementary Table S24), including collagen-containing extracellular matrix (*P *= 1.91 × 10^−6^) and fibroblast migration (*P *= 1.28 × 10^−3^). Protein–protein interaction (PPI) network analysis found many interactions among 33 CVD-associated protein-coding genes ­(Supplementary Fig. S8a), while only the interactions of *THBS4* with *ECM1* and *COL6A3* were identified among 20 CMR-associated protein-coding genes (Supplementary Fig. S8b).

For the union set of protein-coding genes associated with CVDs or CMR traits (49 unique genes), phenotype enrichment analysis highlighted 10 CVD-associated genes (*ACADVL*, *ACP1*, *AGER*, *AGRN*, *AGT*, *ANGPTL4*, *CCN3*, *DSC2*, *FN1*, and *OGN*) and 8 CMR-associated genes (*AGER*, *CCN3*, *COL6A3*, *MANBA*, *PILRA*, *RSPO2*, *SRL*, and *THBS4*) to be associated with cardiovascular system phenotype, with most of other genes associated with certain CVD-related phenotypes (Supplementary Fig. S9a; Supplementary Table S25). The CVD or CMR-associated genes were more related to cardiovascular phenotypes compared with other genes in Mouse Genome Informatics (MGI) platform (32.6% versus 20.1%; *P *= 4.73 × 10^−2^), suggesting the existence of phenotype specificity (Supplementary Fig. S9b; ­Supplementary Table S26).

Besides, for 33 CVD-associated protein-coding genes, 214 drug–gene interactions between 17 protein-coding genes and 202 drugs or components were identified (Supplementary Table S27). Among these, 17 genes in category 1 have been targeted by approved drugs, in which 13 genes (*ACP1*, *AGER*, *AGT*, *APOC3*, *F11*, *FER*, *FN1*, *IL6R*, *KNG1*, *LCT*, *MMP12*, *NCAN*, and *PCSK9*) have also been targeted by drugs currently investigated in clinical trials (category 2). Drugs targeting *AGT* (Quinapril, Enalapril, and Lisinopril, etc.) benefit the treatment of CVDs (HYPTE, MI, and congestive HF, etc.) and renal disease. Of these, 16 genes in category 3 are expected to be further investigated for the treatment of CVDs, such as *ANGPTL4*, *OGN*, and *SPON1*. For 20 CMR-associated protein-coding genes, 66 drug–gene interactions between 8 protein-coding genes and 66 drugs or components were identified (Supplementary Table S28). Among these, eight genes were in category 1 targeted by at least one approved drug, in which six genes (*AGER*, *COL6A3*, *DPEP1*, *FER*, *GSS*, and *HP*) have also been targeted by experimental drugs ­(category 2). Twelve genes in category 3 have not been therapeutic targets.

Single-cell tissue-specific analysis showed differential *FER* expression between AA case group and control group (Wilcoxon Rank Sum test, adjusted *P *= 5.65 × 10^−126^). Cell type-specific analy­sis in eight AA cases found that *FER* was differentially expressed in seven cell types, with high expression in six cell types (smooth muscle cells [SMC1 and SMC3], fibroblast cells, mast cells, endothelial cells, monocytes/macrophages/dendritic cells [Mono/Maph/DC]) and low expression in T lymphocytes (T cells) (Fig. 4). In cell type-specific analysis in three controls, *FER* was highly expressed in mast cells and lowly expressed in T cells (Fig. 4). The findings suggest the distinct patterns of *FER* expression across case group and control group, with two cell types (mast cells and T cells) simultaneously identified in case group and control group, leading to four cell types (SMC, endothelial cells, fibroblast cells, and Mono/Maph/DC cells) highlighted only in case group (Supplementary Figs. S10–S12; Supplementary Table S29).

### Potential side effect of common proteins associated with both CVDs and CMR traits

Agnostic phenome-wide MR (Phe-MR) analysis on 688 binary traits (Supplementary Table S30) identified 14 significant associations, among which 71.4% (10 of 14) were beneficial to other indications (Supplementary Table S31), suggesting that the drugs targeted these proteins may also benefit other diseases or have some off-target risks. Specifically, genetically determined higher level of plasma protein encoded by *AGER* was not only associated with a lower risk of MI but also with a lower risk of other diseases, including hypothyroidism, asthma, hemorrhage of rectum and anus, hematuria, rheumatoid arthritis, and other inflammatory polyarthropathies, however, was associated with a higher risk of multiple sclerosis (MS), intestinal malabsorption (non-celiac), and celiac disease. Genetically higher levels of plasma *CCN3* was associated with a lower risk of MI, as well as diverticulosis and diverticulitis. In addition, genetically predicted higher level of *FER* was associated with a higher risk of AA, VV, and intervertebral disc disorders, ­suggesting that drugs targeting *FER* may also decrease the risk of intervertebral disc disorders. Genetically predicted *SPON1* was associated with decreased risk of chronic ulcer of skin, which were considered deleterious.

## Discussion

In this study, potentially druggable plasma proteins were highlighted for CVDs through comprehensive multi-omics integration analysis incorporating CMR traits. This study not only identified previously reported proteins (*AGER*, *CCN3*, and *SPON1*) but also highlighted novel biologically plausible proteins (*FER*) associated with both CVDs and CMR traits, which could improve the understanding of CVD pathogenesis and identify potential novel therapeutic targets.

Several interesting patterns were found among the potentially causal associations between proteins and CVDs as well as CMR traits. In particular, among 33 CVD-associated proteins, 7 protein-coding genes (*FER*, *IL6R*, *PCSK9*, *ANGPTL4*, *APOC3*, *BMP1*, and *NPNT*) were causally associated with at least two CVDs, while for 20 CMR-associated proteins, 9 protein-coding genes (*COL6A3*, *LRIG1*, *USP15*, *LGALS4*, *FER*, *PILRA*, *SPON1*, *GSS*, and *ECM1*) were causally associated with more than one CMR trait. Of note, *FER* was causally associated with more than one CMR trait and CVD. Notably, several protein–CVD or protein–CMR associations implicated in the study had supportive evidence in either gene polymorphisms, gene expression levels, or protein levels from previous studies ­(Supplementary Table S32).

Notably, there were seven protein–CVD pairs showing opposite effect directions between MR analysis and observational association analysis, for which there are several possible explanations: (i) differences in sample size and statistical power between MR (pQTL data were from 35,559 Iceland participants and CVD GWAS summary data from 377,277 Finland participants) and observational analyses (21,857–34,941 UKB participants, with CVD cases ranging from 283 to 10,454) may contribute to opposite effect ­directions; (ii) population heterogeneity and unmeasured confounding in the observational study may bias the associations; and (iii) MR analyses, though less prone to confounding, only reflect lifelong genetic effects different from short-term exposure effects often captured by observational studies.

Importantly, our study also identified four common proteins with potential pleiotropic effect, which were simultaneously associated with both CVDs and CMR traits, suggesting the shared molecular mechanisms linking cardiovascular structure and function with CVD. *AGER*, also known as *RAGE* (receptor of advanced glycation end products [AGEs]), is a multiligand-binding member of the immunoglobulin superfamily, and is closely related to inflammatory responses. A previous study showed that higher levels of monocyte *RAGE* and lower levels of plasma soluble *RAGE* are linked to higher mortality in cardiogenic shock, a critical complication of acute MI [18]. More importantly, an animal experiment study found that *RAGE* is regulated by HOTAIR, the first long non-coding RNA discovered to regulate gene expression at the transcript level, thereby alleviating inflammatory response in cardiomyocytes after acute MI [19], which is consistent with the findings that higher levels of *AGER* have a protective effect on MI. In addition, *RAGE* is associated with the development of atherosclerosis [20], which could lead to stenosis and possibly further affect cardiac output. An experimental study based on mouse model demonstrated that blocking the *RAGE* axis could increase left ventricular ejection fraction [21], which also suggests an unfavorable effect of the *RAGE* gene on LVCO. While the AGE–RAGE (AGER) axis has been pursued clinically (for example, small-molecule RAGE antagonists such as azeliragon have undergone phase II/III programs, mainly in Alzheimer’s disease [22]), direct and large-scale interventional trials targeting these proteins for cardiovascular endpoints are limited. Moreover, Phe-MR analysis suggested that when developing drugs targeting AGER, heightened vigilance is required regarding the risk of MS, intestinal malabsorption, and celiac disease as potential off-target effects. MS is a demyelinating disorder caused by aberrant activation of immune responses [23], while *AGER* modulates numerous steps of immune response [24]. AGEs, which are bound with AGER, have effects on intestinal health [25].

*CCN3*, which encodes cellular communication network factor 3, is a small secreted cysteine-rich protein. CCN family acts as regulators of the extracellular matrix and *CCN3* inhibits the migration and proliferation of vascular smooth muscle cells [26]. Furthermore, the overexpression of *CCN3* inhibits the inflammation and progression of atherosclerosis in apolipoprotein E-deficient mice [27]. All these processes contribute importantly to AA pathogenesis. An experimental study has shown that matricellular protein CCN3 could serve as a negative regulator of abdominal AA initiation and progression by ameliorating inflammatory cell infiltration and smooth muscle cell apoptosis as well as elastin degradation [28], which is consistent with the protective effect of higher level of *CCN3* on AA implicated in this study. LVESV is a marker of ventricular contractility that is relatively insensitive to loading conditions, and *CCN3* expression has been demonstrated in cardiac muscle [29], which might affect ventricular muscle contraction and thus influence the LVESV. Moreover, CCN3 have been identified as a therapeutic target for insulin-resistant diabetes mellitus in both adults and pediatric patients [30], indicating the potential of CCN3 as a therapeutic target for MI.

*SPON1*, which encodes spondin 1, is a matricellular protein and regulator of macrophage proliferation. Previous studies showed MR evidence of causal relationships between *SPON1* and AF [31, 32] as well as *SPON1* and LVESV [31]. *SPON1* was implicated in an animal-based study as a genetic mediator of cardiac remodeling induced by ischemia-reperfusion [33], suggesting a potential association of *SPON1* with AF. Another experimental study has shown that *SPON1* is significantly upregulated in porcine atria with fibrillation [34]. In addition, a previous MR study has also shown the causal evidence between *SPON1* and AF [32], consistent with our MR results. The close association of AF with LVESV and Ecc_global in terms of physiological mechanisms further demonstrates the potential of *SPON1* as a promising drug target. However, the clinical development targeting *SPON1* has not yet been reported in trial registries, and further validation is required before it can be considered a therapeutic target. Moreover, Phe-MR results suggested that the potential off-target risk of chronic skin ulcers also warrants thorough investigation when *SPON1* is targeted. As a member of the extracellular matrix protein family, SPON1 ­promotes angiogenesis [35], influences signaling pathways of inflammatory mediators such as interleukin-6 (IL-6) [36], and possesses the capacity to regulate cell differentiation and matrix mineralization [37], suggesting its potential role in chronic ulcer of skin.

*FER* encodes a cytosolic non-receptor tyrosine kinase that influences neutrophil chemotaxis and endothelial permeability. This kinase is involved in regulating the phosphorylation of β-conjugated proteins, which is crucial for cadherin association, and also regulates proliferation and migration of vascular smooth muscle cells (VSMCs) [38]. VSMCs are the major component of the vessel wall and have many functions while maintaining the vascular structure, which might further affect the DAo_max_area and DAo_min_area. A recent study has demonstrated that alterations in VSMCs are an important mechanism underlying AA formation [39]. In addition, endothelial cells are in the inner layer of the aorta and also play a major role in maintaining aortic homeostasis. An increasing number of studies have shown that endothelial cell dysfunction is an important factor for AA [39]. *FER* was also implicated in the regulation of inflammation and innate immunity [40], which also plays an important role in the course of AA. In addition, *FER* plays a mechanistic role in vascular and platelet biology [41], and has been exploited as a therapeutic target for numerous antineoplastic agents (dgidb.org/), while no therapies specifically targeting *FER* for CVDs have been registered in clinical trial databases to date. The protective effect of decreased *FER* on AA is reported as a novel finding, although further investigations are needed for validation.

In addition, the druggability exploration showed that 22 proteins associated with CVDs or CMR traits have been targeted by approved or experimental drugs or components, among which six proteins (*AGT*, *APOC3*, *F11*, *FN1*, *MMP12*, and *PCSK9*) have been targeted by drugs with cardiovascular indications. For example, some drugs (chlorthalidone, cilazapril, benazepril, amlodipine, enalapril, and lisinopril) that targeted *AGT* (angiotensinogen) have been approved for the treatment of HYPTE. Several studies revealed that the polymorphism of *AGT* is associated with the risk of essential HYPTE and AF [42], which was also supported in this study. Of note, although four common proteins have not been targeted by drugs with cardiovascular indications, there are potential of these proteins for drug development, especially with respect to the cardioimmunology, which highlights the immune system in cardiac homeostasis and disease [43]. Further experimental studies are warranted to validate the roles of these proteins in CVD mechanisms, thus providing the insights into therapeutic development.

Our study has several strengths. This is the first study to date to investigate the complex associations between a large number of plasma proteins and a comprehensive range of CVDs and CMR traits under systematical analytic framework by leveraging three completely different but mutually complementary methods (PWAS, MR, and Bayesian colocalization), with additional replication ­analyses and a variety of multidimensional downstream analyses. Additional paralleled sensitivity MR analyses integrating ARIC and deCODE pQTL data with CVD GWAS from large scale UKB data also provided further evidence on the protein–CVD pairs with causal relationships (Supplementary Tables S33–S36). Of note, we used the largest plasma pQTL data with imputation model of protein expression. In addition, through incorporating CMR traits into the analysis, our study especially highlighted four proteins simultaneously associated with CVDs and CMR traits. More importantly, additional evidence from druggability evaluation provided more insights into the potential of candidate proteins to be promising druggable targets.

In summary, our findings not only identified plasma proteins causally associated with CVDs or CMR traits but also highlighted the potential of these proteins to be therapeutic targets, which could provide biological insights into CVD pathogenesis and benefit the development of promising preventive and therapeutic drugs.

## Limitations of the study

Our study is not without limitations. First, all analyses were based on European population due to the data accessibility. Therefore, the results cannot be easily generalized to other population unless additional evaluation were undertaken. Second, due to the availability of genetic imputation model of protein expression relied on complete individual-level data (including genotype and protein expression information), only 1348 proteins with significant *cis*-heritability were included, which only accounts for 28.95% of all measurable proteins in ARIC study. The remaining proteins—which may also possess potential druggable relevance for CVDs—could not be evaluated in the present analysis. Future studies may expand this scope as additional genetic imputation models become available, such as those derived from the deCODE Genetics and UKB cohorts. Third, in addition to pQTL mapping, more genetic investigations based on other omics studies, such as eQTL, mQTL, and single-cell sequencing, are needed to offer an improved understanding of the molecular mechanisms implicated in CVDs and CMR traits. In addition, further large-scale longitudinal studies are warranted to account for the dynamic changes of both the protein level and CMR measurements. Finally, MR evidence reflects the effect of lifetime protein exposure and may not be directly translated to the benefits of short-term drug treatment on the risk of CVDs. More importantly, the variability in sample characteristics or measurement techniques across datasets would have substantial influence on the analysis, which needs to be considered in future investigations.

## Materials and methods

### Data sources

#### GWAS summary data of CVDs

GWAS summary data of CVDs were obtained from FinnGen R9 release [44]. A total of 19 common CVDs were included: cardiac arrest (CA), paroxysmal tachycardia (PAR), AF, hypertensive heart disease (HHD), cardiomyopathy (CM), conduction disorders (CDD), non-rheumatic valve diseases (NRV), MI, angina pectoris (AP), dissection of aorta (AD), VV, HF, AA, CAS, PAS, stroke (STR), hypotension (HYPOTE), HYPTE, and venous thromboembolism (VTE), with GWAS sample sizes ranging from 194,232 (2,308 cases and 191,924 controls) to 377,277 (19,372 cases and 357,905 controls). All GWAS data were of European ancestry, with details provided in Supplementary Table S37.

#### GWAS summary data of CMR traits

GWAS summary data of 82 CMR traits were derived from 31,875 subjects in UKB study [45], including 64 left ventricle traits, 4 left atrium traits, 4 right ventricle traits, 4 right atrium traits, 3 ascending aorta traits, and 3 descending aorta traits, which are publicly available via Heart Imaging Genetics Knowledge Portal (Heart-KP), with details provided in Supplementary Table S38.

#### Human plasma pQTL data

Two large-scale plasma pQTL data with European ancestry were used, including ARIC plasma pQTL data for discovery analysis and deCODE genetics pQTL data for external replication analysis. The ARIC plasma pQTL data were based on 7213 European American individuals [46], with protein imputation models for 1348 plasma proteins with significant nonzero *cis*-heritability (*P *< 0.01) generated by elastic net models. The deCODE plasma pQTL data involved 27.2 million genetic variants and 4907 plasma protein SOMAmers of 35,559 Icelanders [47]. Given that *cis*-pQTLs were considered to have a more direct and specific biological effect on protein, we used the publicly available *cis*-pQTLs for discovery analysis and extracted the *cis*-regions around ±1 Mb of the transcription start site of protein-coding genes for external replication analysis.

#### Single-cell RNA sequencing data

Single-cell RNA sequencing (scRNA-seq) data used in this study were retrieved from the Gene Expression Omnibus (GEO) database under accession number GSE155468. This dataset comprises single-cell transcriptomic profiles generated from 11 ascending aortic tissue samples, including 8 aneurysmal ascending aortic wall tissues collected from patients diagnosed with ascending thoracic aortic aneurysm and 3 non-diseased ascending aortic wall tissue from non-aneurysmal control individuals (2 heart transplant recipients and 1 lung donor) [48]. The original study performed tissue dissociation followed by single-cell sequencing, providing high-resolution characterization of cellular heterogeneity within the aortic wall tissues. A total of 48,128 qualified cells were obtained for further analysis.

### Statistical analysis

#### Discovery analysis

We initially implemented PWAS, MR, and colocalization analyses to sequentially screen the potential protein–CVD and protein–CMR pairs with causal relationship. All analyses were performed with the major histocompatibility complex region excluded due to its complex linkage disequilibrium (LD) structure. Details of these methods are provided (Supplementary Methods). We performed two parallel PWAS analyses integrating the available imputation models of 1348 *cis*-heritable plasma proteins derived from ARIC study with the GWAS of 19 CVDs and 82 CMR traits, respectively, to identify the protein–CVD and protein–CMR associations. We used in-sample LD reference data from individuals of European ancestry to account for LD. Given that multiple non-independent tests may increase false positive findings and the Bonferroni correction is often too stringent, we employed Benjamini–Hochberg FDR method for multiple testing correction on PWAS and the following main analysis results of each CVD and CMR trait [49], which enabled a more reliable, rigorous interpretation of the study results. The significance level declared as FDR-corrected *P* value < 0.05.

Additionally, we performed TWAS using the FUSION framework [50] to integrate CVD and CMR GWAS with multi-tissue expression predictive models from GTEx v8 (Genotype-Tissue Expression project). These models were constructed for five cardiovascular-relevant tissues, including aorta, coronary artery, atrial appendage, left ventricle, and whole blood. We used LD reference data from the 1000 Genomes Project European-ancestry panel and considered *P* < 0.05 as the significance threshold to explore whether PWAS-significant protein–CVD and protein–CMR pairs showed consistent evidence at the transcript level. We considered proteins associated with CVD or CMR traits to be TWAS-significant if they are associated with at least one of five cardiovascular-relevant tissues.

To investigate the potential causal relationships between PWAS-significant proteins and CVDs or CMR traits, we performed two-sample MR analysis together with various sensitivity analyses. The MR analysis was conducted and is reported in accordance with the STROBE-MR Statement [51] (Supplementary Table S39 ), which mainly involves instrumental variable (IV) selection, IV assessment, primary MR analysis, and sensitivity analysis. For IV selection, we firstly used a protein-specific Bonferroni-corrected *P*-value threshold (0.05/the number of SNPs in *cis*-region) to define significant pQTLs and obtained the independent *cis*-pQTLs (*r*^2^ < 0.01 in the 1-Mb *cis*-region) for each protein. Then, we harmonized the alleles of IVs, removed palindromic SNPs with ambiguous allele frequencies (0.42–0.58), and also performed additional sensitivity MR analyses by retaining all palindromic SNPs. For IV assessment, we estimated *R*^2^ to quantify the proportion of exposure variance explained by the IVs and further used *F*-statistics to assess instrument strength, with empirical threshold of *F*-statistics larger than 10 indicating that MR estimates would be less affected by weak instrument bias. In the primary MR analysis, the Wald ratio method [52] was used for proteins with only one IV, the fixed-effect inverse-variance weighted (IVW) method [53] for proteins with two or three IVs, and the random-effect IVW method [54] for proteins with four or more IVs, as heterogeneity increases with the number of IVs. Because the random-effect model could account for heterogeneity across IVs by allowing for over-dispersion of the regression model, we retained the results even in the presence of the heterogeneity. We also performed additional analyses including MR-Egger [55] and weighted median [56] to account for horizontal pleiotropy when more than three IVs were available. Given that MR-Egger allows for the correction of horizontal pleiotropy, we prioritized its estimates over those from the IVW method for the associations with significant horizontal pleiotropy detected, and only associations that remained statistically significant in the MR-Egger analysis were retained. For significant protein–CVD and protein–CMR pairs identified in primary MR analysis, further sensitivity analyses, including MR-Egger, heterogeneity test, MR Steiger, and leave-one-out, were also conducted to assess the robustness of the results (Supplementary Methods). All MR analyses were performed using the MendelianRandomization 0.9.0 package in R version 4.2.1, and TwoSampleMR 0.5.6 package was used to conduct sensitivity analysis.

To investigate whether protein expression and CVDs or CMR traits are driven by shared causal variants, we performed ­colocalization analysis [10] for the potential causal protein–CVD or protein–CMR pairs identified by MR using the R package coloc (version 5.2.2) under the default setting (*p*_1_ = *p*_2_ = 1 × 10^−4^, *p*_12_ = 1 × 10^−5^) (Supplementary Methods). We mainly focused on the PP.H4 in the colocalization analysis, which reflects the case that protein expression and trait share the same causal SNP. We conducted colocalization analysis using ARIC *cis*-pQTL data (within 500 kb of the transcription start site of protein-coding genes), with PP.H4 ≥ 0.7 [57, 58] defined as a strong evidence of colocalization.

#### External replication and observational association analysis

For the significant protein–CVD and protein–CMR pairs screened by discovery analysis, we further performed replication MR analysis using the same stringent procedures for external replication by integrating the CVD and CMR GWASs with deCODE genetics pQTL data to assess the consistency and robustness of the causal associations.

For significant protein–CVD and protein–CMR associations passed external replication analysis, we further examined the associations between baseline protein levels and CVDs or CMR traits using individual level data from UKB (Supplementary Methods). Specifically, we obtained the normalized protein expression data for 2923 proteins of 53,058 participants from UKB, using Cox regression to explore the associations between proteins and CVD risk. We also obtained individual CMR measurement data of 39,698 individuals from UKB research analysis platform, using linear regression to estimate the protein–CMR associations.

#### Multidimensional downstream analyses

For proteins significantly associated with CVDs or CMR traits and passed external replication analysis, we performed multidimensional downstream analyses. We first performed enrichment ­analysis using R package clusterProfiler 4.8.3 under default setting to explore underlying biological pathways. Here, we mainly focused on the biological processes in GO and pathways in KEGG. We declared the significant terms or pathways at FDR corrected *P *< 0.05. In addition, using the STRING database [59], we generated the PPI network to display the potential interrelationships among the significant proteins, with the minimum required interaction score set at 0.4.

In addition, we performed phenotype enrichment analysis based on the existing mouse/human orthology with phenotype annotations from the MGI platform to explore the gene-associated phenotypes. We further characterized the phenotype specificity of the CVD or CMR-associated genes against the non-associated genes, by examining the differences in the proportions of genes associated with certain phenotypes in the CVD or CMR-associated gene group against that in the non-associated gene group using Fisher exact test.

We searched the proteins passed the external replication in the Drug-Gene Interaction Database (DGIdb), DrugBank, and ChEMBL databases to prioritize the potential druggable targets. According to the drug information and the clinical trials of drugs which targeted the proteins, we classified the proteins into three categories: (i) approved (already served as therapeutic targets); (ii) druggable (reported in clinical trials); and (iii) untargeted (no current druggable evidence).

For proteins simultaneously associated with multiple CVDs and CMR traits, we performed tissue-specific and cell type-specific single-cell gene expression analysis to examine the patterns of differential expression across cases and controls using scRNA-seq data and Seurat 4.4.0 package [60] in R. Specifically, we firstly removed the genes detected in fewer than three cells and the cells with gene counts of less than 200 or more than 5000, or with more than 10% mitochondrial genes. We then integrated all samples, performed clustering, and annotated different cell types based on the original study, Cellmarker 2.0 [61], and previously reported gene markers. We subsequently used “Findmarkers” function to examine whether the interesting genes were differentially expressed between case group and control group using Wilcoxon rank sum test. Furthermore, we reanalyzed the cell type-specific differential expression between cases and controls to identify the specific cell types where these genes are differentially expressed and to compare these cell types between the two groups. We followed the same criteria and process to integrate data, find clusters, and annotate cell types. We then used the “FindAllmarkers” function with Wilcoxon rank sum test to identify marker genes for each cell type. We defined the significance as Bonferroni-corrected (function default) *P *< 0.01 and average log_2_Fold Change (avg_log_2_FC) > 0.1.

Finally, Phe-MR analysis was performed to further assess the potential side effects for common proteins associated with both CVDs and CMR traits. GWAS of 1409 binary traits were obtained from UKB cohort [62], and phenotypes with fewer than 500 cases were excluded to ensure statistical power, leading to a total of 688 non-CVD phenotypes included. pQTLs used in Phe-MR analysis were derived from the ARIC study. The findings from Phe-MR ­analysis could be interpreted as the risk/protective effect per ­standard deviation (per-SD) increase in the plasma protein level. Specifically, if the effect direction of plasma proteins on certain indication and CVDs were both negative, the identified protein that showed protective effect on CVDs may also be beneficial for these indications, and *vice versa*.

## Supplementary Material

loag001_Supplementary_Data

## Data Availability

The GWAS summary data of CVDs can be accessed from FinnGen (finngen.fi/en/access_results), and GWAS of CMR traits were obtained from Heart-KP (heartkp.org). Two pQTL data are available from ARIC (nilanjanchatterjeelab.org/pwas) and deCODE genetics (decode.com/summarydata). Multi-tissue expression predictive models for five cardiovascular-relevant tissues from GTEx project v8 (gusevlab.org/projects/fusion) were utilized. The individual level data were obtained from UK Biobank under application no. 88159. We used DGIdb (dgidb.org), DrugBank (go.drugbank.com), and ChEMBL (ebi.ac.uk/chembl) platforms to prioritize the druggable targets. The scRNA-seq data can be downloaded from GEO (ncbi.nlm.nih.gov/geo).

## References

[1] Mensah GA , Fuster V, Murray CJL et al Global burden of cardiovascular diseases and risks, 1990-2022. J Am Coll Cardiol 2023;82:2350–473. 38092509 10.1016/j.jacc.2023.11.007 PMC7615984

[2] Townsend N , Kazakiewicz D, Lucy Wright F et al Epidemiology of cardiovascular disease in Europe. Nat Rev Cardiol 2022;19:133–43. 34497402 10.1038/s41569-021-00607-3

[3] Flora GD , Nayak MK. A brief review of cardiovascular diseases, associated risk factors and current treatment regimes. Curr Pharm Des 2019;25:4063–84. 31553287 10.2174/1381612825666190925163827 PMC12994374

[4] Tsao CW , Aday AW, Almarzooq ZI et al Heart Disease and Stroke Statistics-2023 update: a report from the American Heart Association. Circulation 2023;147:e93–e621. 36695182 10.1161/CIR.0000000000001123 PMC12135016

[5] Fordyce CB , Roe MT, Ahmad T et al Cardiovascular drug ­development: is it dead or just hibernating? J Am Coll Cardiol 2015;65:1567–82. 25881939 10.1016/j.jacc.2015.03.016

[6] Palstrom NB , Matthiesen R, Rasmussen LM et al Recent ­developments in clinical plasma proteomics-applied to cardiovascular research. Biomedicines 2022;10:162. 35052841 10.3390/biomedicines10010162 PMC8773619

[7] Santos R , Ursu O, Gaulton A et al A comprehensive map of molecular drug targets. Nat Rev Drug Discov 2017;16:19–34. 27910877 10.1038/nrd.2016.230 PMC6314433

[8] Wingo AP , Liu Y, Gerasimov ES et al Integrating human brain proteomes with genome-wide association data implicates new proteins in Alzheimer’s disease pathogenesis. Nat Genet 2021;53:143–6. 33510477 10.1038/s41588-020-00773-z PMC8130821

[9] Haycock PC , Burgess S, Wade KH et al Best (but oft-forgotten) practices: the design, analysis, and interpretation of Mendelian randomization studies. Am J Clin Nutr 2016;103:965–78. 26961927 10.3945/ajcn.115.118216 PMC4807699

[10] Giambartolomei C , Vukcevic D, Schadt EE et al Bayesian test for colocalisation between pairs of genetic association studies using summary statistics. PLoS Genet 2014; 10: e1004383. 24830394 10.1371/journal.pgen.1004383 PMC4022491

[11] Rasooly D , Peloso GM, Pereira AC et al Genome-wide association analysis and Mendelian randomization proteomics identify drug targets for heart failure. Nat Commun 2023;14:3826. 37429843 10.1038/s41467-023-39253-3 PMC10333277

[12] Schmidt AF , Bourfiss M, Alasiri A et al Druggable proteins influencing cardiac structure and function: implications for heart failure therapies and cancer cardiotoxicity. Sci Adv 2023;9:eadd4984. 37126556 10.1126/sciadv.add4984 PMC10132758

[13] Brandes N , Linial N, Linial M. PWAS: proteome-wide association study-linking genes and phenotypes by functional variation in proteins. Genome Biol 2020;21:173. 32665031 10.1186/s13059-020-02089-x PMC7386203

[14] Bai W , Suzuki H, Huang J et al A population-based phenome-wide association study of cardiac and aortic structure and function. Nat Med 2020;26:1654–62. 32839619 10.1038/s41591-020-1009-y PMC7613250

[15] Pirruccello JP , Bick A, Wang M et al Analysis of cardiac magnetic resonance imaging in 36,000 individuals yields genetic insights into dilated cardiomyopathy. Nat Commun 2020;11:2254. 32382064 10.1038/s41467-020-15823-7 PMC7206184

[16] Ponikowski P , Voors AA, Anker SD et al 2016 ESC Guidelines for the diagnosis and treatment of acute and chronic heart failure: the Task Force for the diagnosis and treatment of acute and chronic heart failure of the European Society of Cardiology (ESC) developed with the special contribution of the Heart Failure Association (HFA) of the ESC. Eur Heart J 2016;37:2129–200. 27206819 10.1093/eurheartj/ehw128

[17] Armstrong AC , Gidding S, Gjesdal O et al LV mass assessed by echocardiography and CMR, cardiovascular outcomes, and medical practice. JACC Cardiovasc Imaging 2012;5:837–48. 22897998 10.1016/j.jcmg.2012.06.003 PMC3501209

[18] Selejan SR , Hewera L, Hohl M et al Suppressed MMP-9 activity in myocardial infarction-related cardiogenic shock implies diminished rage degradation. Shock 2017;48:18–28. 28608784 10.1097/SHK.0000000000000829

[19] Lu W , Zhu L, Ruan ZB et al HOTAIR promotes inflammatory response after acute myocardium infarction by upregulating RAGE. Eur Rev Med Pharmacol Sci 2018;22:7423–30. 30468490 10.26355/eurrev_201811_16282

[20] Hudson BI , Lippman ME. Targeting RAGE signaling in inflammatory disease. Annu Rev Med 2018;69:349–64. 29106804 10.1146/annurev-med-041316-085215

[21] Wasim R , Mahmood T, Siddiqui MH et al Entanglement of AGE-RAGE axis in cardiac pathosis. *bioRxiv.* 2023. Doi: 10.1101/2023.07.23.550244

[22] Burstein AH , Sabbagh M, Andrews R et al Development of azeliragon, an oral small molecule antagonist of the receptor for advanced glycation endproducts, for the potential slowing of loss of cognition in mild Alzheimer’s disease. J Prev Alzheimers Dis 2018;5:149–54. 29616709 10.14283/jpad.2018.18 PMC12280790

[23] Taheri M , Nemati S, Movafagh A et al TRAIL gene expression analysis in multiple sclerosis patients. Hum Antibodies 2016;24:33–8. 27472871 10.3233/HAB-160291

[24] Chavakis T , Bierhaus A, Nawroth PP. RAGE (receptor for advanced glycation end products): a central player in the inflammatory response. Microbes Infect 2004;6:1219–25. 15488742 10.1016/j.micinf.2004.08.004

[25] Nie C , Li Y, Qian H et al Advanced glycation end products in food and their effects on intestinal tract. Crit Rev Food Sci Nutr 2022;62:3103–15. 33356474 10.1080/10408398.2020.1863904

[26] Shimoyama T , Hiraoka S, Takemoto M et al CCN3 inhibits neointimal hyperplasia through modulation of smooth muscle cell growth and migration. Arterioscler Thromb Vasc Biol 2010;30:675–82. 20139355 10.1161/ATVBAHA.110.203356

[27] Liu J , Ren Y, Kang L et al Overexpression of CCN3 inhibits inflammation and progression of atherosclerosis in apolipoprotein E-deficient mice. PLoS One 2014; 9: e94912. 24722330 10.1371/journal.pone.0094912 PMC3983261

[28] Fingerlin TE , Zhang W, Yang IV et al Genome-wide imputation study identifies novel HLA locus for pulmonary fibrosis and potential role for auto-immunity in fibrotic idiopathic interstitial pneumonia. BMC Genet 2016;17:74. 27266705 10.1186/s12863-016-0377-2 PMC4895966

[29] Lin Z , Natesan V, Shi H et al A novel role of CCN3 in regulating endothelial inflammation. J Cell Commun Signal 2010;4:141–53. 21063504 10.1007/s12079-010-0095-x PMC2948121

[30] Paradis R , Lazar N, Antinozzi P et al Nov/Ccn3, a novel transcriptional target of FoxO1, impairs pancreatic β-cell function. PLoS One 2013;8:e64957. 23705021 10.1371/journal.pone.0064957 PMC3660386

[31] Shah AM , Myhre PL, Arthur V et al Large scale plasma proteomics identifies novel proteins and protein networks associated with heart failure development. Nat Commun 2024;15:528. 38225249 10.1038/s41467-023-44680-3 PMC10789789

[32] Wang Q , Richardson TG, Sanderson E et al A phenome-wide bidirectional Mendelian randomization analysis of atrial fibrillation. Int J Epidemiol 2022;51:1153–66. 35292824 10.1093/ije/dyac041 PMC9365635

[33] Barallobre-Barreiro J , Didangelos A, Schoendube FA et al Proteomics analysis of cardiac extracellular matrix remodeling in a porcine model of ischemia/reperfusion injury. Circulation 2012;125:789–802. 22261194 10.1161/CIRCULATIONAHA.111.056952

[34] Chen CL , Lin JL, Lai LP et al Altered expression of FHL1, CARP, TSC-22 and P311 provide insights into complex transcriptional regulation in pacing-induced atrial fibrillation. Biochim Biophys Acta 2007;1772:317–29. 17174532 10.1016/j.bbadis.2006.10.017

[35] Chang H , Dong T, Ma X et al Spondin 1 promotes metastatic progression through Fak and Src dependent pathway in human osteosarcoma. Biochem Biophys Res Commun 2015;464:45–50. 26032498 10.1016/j.bbrc.2015.05.092

[36] Huo Y , Yang J, Zheng J et al Increased SPON1 promotes pancreatic ductal adenocarcinoma progression by enhancing IL-6 trans-signalling. Cell Prolif 2022; 55: e13237. 35487760 10.1111/cpr.13237 PMC9136514

[37] Palmer GD , Piton AH, Thant LM et al F-spondin regulates chondrocyte terminal differentiation and endochondral bone formation. J Orthop Res 2010;28:1323–9. 20839318 10.1002/jor.21130 PMC3245523

[38] Lyon C , Mill C, Tsaousi A et al Regulation of VSMC behavior by the cadherin-catenin complex. Front Biosci (Landmark Ed) 2011;16:644–57. 21196194 10.2741/3711

[39] Gao J , Cao H, Hu G et al The mechanism and therapy of aortic aneurysms. Signal Transduct Target Ther 2023;8:55. 36737432 10.1038/s41392-023-01325-7 PMC9898314

[40] Greer P. Closing in on the biological functions of Fps/Fes and Fer. Nat Rev Mol Cell Biol 2002;3:278–89. 11994747 10.1038/nrm783

[41] Lennartsson J , Ma H, Wardega P et al The Fer tyrosine kinase is important for platelet-derived growth factor-BB-induced signal transducer and activator of transcription 3 (STAT3) protein phosphorylation, colony formation in soft agar, and tumor growth *in vivo*. J Biol Chem 2013;288:15736–44. 23589302 10.1074/jbc.M113.476424 PMC3668732

[42] Fajar JK , Pikir BS, Sidarta EP et al The genes polymorphism of angiotensinogen (AGT) M235T and AGT T174M in patients with essential hypertension: a meta-analysis. Gene Rep 2019;16:100421.

[43] Swirski FK , Nahrendorf M. Cardioimmunology: the immune system in cardiac homeostasis and disease. Nat Rev Immunol 2018;18:733–44. 30228378 10.1038/s41577-018-0065-8

[44] Kurki MI , Karjalainen J, Palta P et al FinnGen provides genetic insights from a well-phenotyped isolated population. Nature 2023;613:508–18. 36653562 10.1038/s41586-022-05473-8 PMC9849126

[45] Zhao B , Li T, Fan Z et al Heart-brain connections: phenotypic and genetic insights from magnetic resonance images. Science 2023;380:abn6598. 37262162 10.1126/science.abn6598 PMC11987082

[46] Zhang J , Dutta D, Kottgen A et al Plasma proteome analyses in individuals of European and African ancestry identify cis-pQTLs and models for proteome-wide association studies. Nat Genet 2022;54:593–602. 35501419 10.1038/s41588-022-01051-w PMC9236177

[47] Ferkingstad E , Sulem P, Atlason BA et al Large-scale integration of the plasma proteome with genetics and disease. Nat Genet 2021;53:1712–21. 34857953 10.1038/s41588-021-00978-w

[48] Li Y , Ren P, Dawson A et al Single-cell transcriptome analysis reveals dynamic cell populations and differential gene expression patterns in control and aneurysmal human aortic tissue. Circulation 2020;142:1374–88. 33017217 10.1161/CIRCULATIONAHA.120.046528 PMC7539140

[49] Glickman ME , Rao SR, Schultz MR. False discovery rate control is a recommended alternative to Bonferroni-type adjustments in health studies. J Clin Epidemiol 2014;67:850–7. 24831050 10.1016/j.jclinepi.2014.03.012

[50] Gusev A , Ko A, Shi H et al Integrative approaches for large-scale transcriptome-wide association studies. Nat Genet 2016;48:245–52. 26854917 10.1038/ng.3506 PMC4767558

[51] Skrivankova VW , Richmond RC, Woolf BAR et al Strengthening the reporting of observational studies in epidemiology using Mendelian randomisation (STROBE-MR): explanation and elaboration. BMJ 2021;375:n2233. 34702754 10.1136/bmj.n2233 PMC8546498

[52] Burgess S , Small DS, Thompson SG. A review of instrumental variable estimators for Mendelian randomization. Stat Methods Med Res 2017;26:2333–55. 26282889 10.1177/0962280215597579 PMC5642006

[53] Burgess S , Butterworth A, Thompson SG. Mendelian randomization analysis with multiple genetic variants using summarized data. Genet Epidemiol 2013;37:658–65. 24114802 10.1002/gepi.21758 PMC4377079

[54] Bowden J , Del Greco MF, Minelli C et al A framework for the investigation of pleiotropy in two-sample summary data ­Mendelian randomization. Stat Med 2017;36:1783–802. 28114746 10.1002/sim.7221 PMC5434863

[55] Bowden J , Davey Smith G, Burgess S. Mendelian randomization with invalid instruments: effect estimation and bias detection through Egger regression. Int J Epidemiol 2015;44:512–25. 26050253 10.1093/ije/dyv080 PMC4469799

[56] Bowden J , Davey Smith G, Haycock PC et al Consistent estimation in Mendelian randomization with some invalid instruments using a weighted median estimator. Genet Epidemiol 2016;40:304–14. 27061298 10.1002/gepi.21965 PMC4849733

[57] Zhu Z , Zhu X, Liu CL et al Shared genetics of asthma and mental health disorders: a large-scale genome-wide cross-trait analysis. Eur Respir J 2019;54:1901507. 31619474 10.1183/13993003.01507-2019

[58] Chen J , Ruan X, Sun Y et al Multi-omic insight into the molecular networks of mitochondrial dysfunction in the pathogenesis of inflammatory bowel disease. EBioMedicine 2024;99:104934. 38103512 10.1016/j.ebiom.2023.104934 PMC10765009

[59] Szklarczyk D , Gable AL, Lyon D et al STRING v11: protein-protein association networks with increased coverage, supporting functional discovery in genome-wide experimental datasets. Nucleic Acids Res 2019;47:D607–D613. 30476243 10.1093/nar/gky1131 PMC6323986

[60] Hao Y , Hao S, Andersen-Nissen E et al Integrated analysis of multimodal single-cell data. Cell 2021;184:3573–87.e29. 34062119 10.1016/j.cell.2021.04.048 PMC8238499

[61] Hu C , Li T, Xu Y et al CellMarker 2.0: an updated database of manually curated cell markers in human/mouse and web tools based on scRNA-seq data. Nucleic Acids Res 2023;51:D870–6. 36300619 10.1093/nar/gkac947 PMC9825416

[62] Zhou W , Nielsen JB, Fritsche LG et al Efficiently controlling for case-control imbalance and sample relatedness in large-scale genetic association studies. Nat Genet 2018;50:1335–41. 30104761 10.1038/s41588-018-0184-y PMC6119127

