# Supplementary material for: Integrative omics analysis incorporating cardiovascular magnetic resonance imaging pinpoints potentially druggable plasma proteins for cardiovascular diseases
Source: Life Metab. 2026 Jan 7;5(3):loag001. doi: 10.1093/lifemeta/loag001 (PMC13128259; doi:10.1093/lifemeta/loag001)
Supplement: loag001_Supplementary_Data [file loag001_supplementary_data.zip › Supplementary_Information - tu - shen-CLEAN.docx]

## Supplementary Methods and Figures

**Integrative omics analysis incorporating cardiovascular magnetic resonance imaging pinpoints potentially druggable plasma proteins for cardiovascular diseases**

Weiming Gong, Ping Guo, Lu Liu, Xiubin Sun, Shukang Wang, Fuzhong Xue, Lujia Shen, Zhongshang Yuan

### Proteome‑wide association studies (PWAS)

PWAS aims to integrate genetic imputation model of protein expression with genome-wide association studies (GWAS) summary statistics to identify the associations between proteins and complex diseases [1]. Using the FUSION pipeline [2], we performed two parallel PWAS analyses combining the available imputation weights of 1,348 significant *cis*-heritable plasma proteins [2] with the GWAS of 19 cardiovascular diseases (CVDs) and 82 cardiovascular magnetic resonance imaging (CMR) traits, respectively. We first calculated the linear sum of imputation weights multiplied by z-score and then performed an association analysis. We used the accompanying in-sample linkage disequilibrium (LD) reference data of European ancestry to account for LD. In addition, the major histocompatibility complex (MHC) region was excluded from the analysis due to its structural complexity. False discovery rate (FDR) correction based on Benjamini-Hochberg (BH) method was used for PWAS results of each CVD and CMR trait, with significance level set at FDR-corrected *P* < 0.05.

### Transcriptome-wide association studies (TWAS)

TWAS is a powerful method integrating expression quantitative trait locus (eQTL) data with GWAS to identify genes associated with complex traits [3]. In general, the promising target genes were more likely to have both PWAS and TWAS signals. Therefore, we explored whether the PWAS-significant protein-CVD and protein-CMR pairs showed consistent evidence at the transcript level. We integrated the CVD and CMR GWASs with multi-tissue expression predictive models of five cardiovascular-relevant tissues from GTEx project v8 (including aorta, coronary, atrial appendage, left ventricle, and whole blood from gusevlab.org/projects/fusion/) to perform TWAS following the FUSION workflow [3], with LD reference data from 1000 Genomes Project European ancestry used. The significance level of TWAS analysis was declared as *P* < 0.05.

### Mendelian randomization (MR)

MR uses genetic variants as instruments to infer potential causal association between exposure and outcome of interest [4]. MR estimates are more closely to reflect underlying causal relationships and are less prone to bias such as selection bias, reverse causality, and confounding, given that the genetic variants used as the instrument are randomized at meiosis. Nevertheless, there are three critical assumptions for instrumental variants used in MR analysis: (ⅰ) relevance assumption: the instrumental variables (IVs) of the exposure should be strongly associated with the exposure; (ⅱ) independence assumption: the IVs of exposure should be independent of confounding factors; (ⅲ) exclusion restriction assumption: the IVs only influence the outcome by the path of exposure. When the IVs satisfy the above assumptions, Wald ratio method [5] (for one IV) or inverse variance weighted method [6, 7] (for multiple IVs, since the potential heterogeneity is more likely to be existed as the number of IVs increases) yields accurate causal estimates. However, these assumptions may be violated sometimes, thus we conducted some sensitivity analyses to ensure the robustness of the MR results.

For significant protein-CMR and protein-CVD pairs identified in primary MR analysis, we conducted several sensitivity analyses. Specifically, we used the intercept term from MR-Egger [8] (*P* < 0.05) as an indicator of horizontal pleiotropy, and then calculated Cochran’s *Q* statistic to assess heterogeneity for proteins with more than three IVs, with *P* < 0.05 indicating the presence of heterogeneity. We further performed MR Steiger directionality test to assess whether the MR analysis was biased by reverse causation and leave-one-out method to test whether the MR estimates were dominantly driven by one IV.

### Bayesian colocalization analysis

The colocalization analysis (coloc) [9] investigates whether protein expression and phenotype are driven by a shared genetic variant. Under the assumption that each trait has at most one causal variant in a locus, coloc enumerates five mutually exclusive configuration models of causal variant: (ⅰ) H0: no causal variant for either protein or phenotype; (ⅱ) H1: one causal variant for protein but no causal variant for phenotype; (ⅲ) H2: one causal variant for phenotype but no causal variant for protein; (4) H3: protein and phenotype have distinct causal variants; (5) H4: protein and phenotype share the same causal variant. Posterior probabilities (PP) were calculated to quantify the support for each model hypothesis (PP.H0, PP.H1, PP.H2, PP.H3, and PP.H4), respectively. When the posterior probability supports H4, it indicates that protein expression and phenotype are affected by the same SNP. We used the default setting (*p*_1_ = *p*_2_ = 1 × 10^−4^, *p*_12_ = 1 × 10^−5^) for coloc and mainly focused on the PP.H4, which reflects the case that protein expression and trait share the same causal SNP. We conducted colocalization analysis using ARIC *cis*-pQTL data (within 500 kb of the transcription start site of protein-coding genes), with PP.H4 ≥ 0.7 [10, 11] defined as a strong evidence of colocalization.

### Observational association analysis

We examined the associations between baseline protein level and CVDs or CMR traits using individual level data from UK Biobank (UKB) [12], which are subjected to stringent data preprocessing. Specifically, we obtained the normalized protein expression data for 2,923 proteins of 53,058 participants from UKB ([biobank.ndph.ox.ac.uk/showcase/label.cgi?id=1839](https://biobank.ndph.ox.ac.uk/showcase/label.cgi?id=1839)). We selected the individuals with British or Irish ancestry based on the self-reported ethnicity and their baseline protein abundance measurement. To further alleviate the impact of confounding, we determined several baseline characteristics as covariates, including age at recruitment, sex, body mass index (BMI; 4 categories: < 25, 25−29, 30−34, > 35 kg/m^2^), alcohol intake frequency, and smoke status. Diagnoses of CVD incident were based on ICD-10 codes available in UKB as that in FinnGen study (Supplementary Table S37). Dates of diagnoses were obtained from the record in UKB. The follow-up time was determined from the date of recruitment to the date of first diagnosis of CVD or to the censoring date. Individuals with missing data and CVD cases that were recorded at baseline were excluded. The sample size for protein-CVD association analysis ranged from 21,857 to 34,941. NPX data were subjected to inverse rank normal transformation. Cox regression models were used to explore the associations between proteins and CVD risk, adjusting for covariates using ‘survival’ R package (Version 3.5-8). In addition, we obtained individual CMR measurement data of 39,698 individuals from UKB research analysis platform ([ukbiobank.dnanexus.com/landing](https://ukbiobank.dnanexus.com/landing)). After selecting the individuals with proteins and covariates data and further excluding the individuals with CVDs or missing data, the sample size for protein-CMR association analysis ranged from 2,189 to 3,303. Data were subjected to inverse rank normal transformation. We estimated the protein-CMR associations using multivariable linear regression models, adjusting for the same covariates mentioned above, using ‘stats’ R package (Version 4.3.2). The significance level was set as FDR-corrected *P* < 0.05.

### Sensitivity MR analyses using UKB CVD GWAS dataset

For protein-CVD associations included in discovery and replication MR analyses, we performed additional sensitivity analyses by integrating plasma pQTL data from ARIC and deCODE with CVD GWAS from UKB [13-15] to serve as parallel sensitivity analyses for the discovery and replication MR analyses, respectively. Specifically, we performed MR analysis using the same stringent procedures by integrating the UKB CVD GWAS with ARIC and deCODE pQTL data for protein-CVD associations included in discovery and replication MR analyses. The significance level was defined as FDR-corrected *P* value < 0.05.

We totally obtained 12 CVD GWAS data from UKB with sample sizes varying from 361,194 (1606 cases and 359,588 controls) to 420,531 (280 cases and 420,251 controls). In sensitivity MR analysis of discovery MR results, a total of 66 protein-CVD pairs were identified, involving 54 unique proteins associated with at least 1 of 9 CVDs (Supplementary Tables S33 and S34). Of these, 61 protein-CVD pairs were overlapped with the discovery MR results. In sensitivity MR analysis for replication MR results, a total of 21 protein-CVD pairs were identified, involving 13 unique proteins associated with at least 1 of 6 CVDs (Supplementary Tables S35−36). Of these, 17 protein-CVD pairs were overlapped with the discovery MR results.

Further, targeting on the overlapped protein-CVD pairs, we found strong correlations between the causal estimates from the analysis using FinnGen CVD GWAS data and that from the analysis using UKB CVD GWAS data (ARIC-FinnGen and ARIC-UKB: *r* = 0.918, *P* < 2.2 × 10^−16^; deCODE-FinnGen and deCODE-UKB: 0.939, *P* < 2.2 × 10^−16^), and the causal effect directions for specific protein-CVD pair derived from these two CVD GWAS data were totally consistent.

**References**

[1] Brandes N, Linial N, Linial M. PWAS: proteome-wide association study-linking genes and phenotypes by functional variation in proteins. *Genome Biol* 2020; **21**:173.

[2] Zhang J, Dutta D, Kottgen A *et al*. Plasma proteome analyses in individuals of European and African ancestry identify cis-pQTLs and models for proteome-wide association studies. *Nat Genet* 2022; **54**:593-602.

[3] Gusev A, Ko A, Shi H *et al*. Integrative approaches for large-scale transcriptome-wide association studies. *Nat Genet* 2016; **48**:245-252.

[4] Sanderson E, Glymour MM, Holmes MV *et al*. Mendelian randomization. *Nat Rev Methods Primers* 2022; **2**:6.

[5] Burgess S, Small DS, Thompson SG. A review of instrumental variable estimators for Mendelian randomization. *Stat Methods Med Res* 2017; **26**:2333-2355.

[6] Burgess S, Butterworth A, Thompson SG. Mendelian randomization analysis with multiple genetic variants using summarized data. *Genet Epidemiol* 2013; **37**:658-665.

[7] Bowden J, Del Greco MF, Minelli C *et al*. A framework for the investigation of pleiotropy in two-sample summary data Mendelian randomization. *Stat Med* 2017; **36**:1783-1802.

[8] Bowden J, Davey Smith G, Burgess S. Mendelian randomization with invalid instruments: effect estimation and bias detection through Egger regression. *Int J Epidemiol* 2015; **44**:512-525.

[9] Giambartolomei C, Vukcevic D, Schadt EE *et al*. Bayesian test for colocalisation between pairs of genetic association studies using summary statistics. *PLoS Genet* 2014; **10**:e1004383.

[10] Zhu Z, Zhu X, Liu CL *et al*. Shared genetics of asthma and mental health disorders: a large-scale genome-wide cross-trait analysis. *Eur Respir J* 2019; **54**:1901507.

[11] Chen J, Ruan X, Sun Y *et al*. Multi-omic insight into the molecular networks of mitochondrial dysfunction in the pathogenesis of inflammatory bowel disease. *EBioMedicine* 2024; **99**:104934.

[12] Bycroft C, Freeman C, Petkova D *et al*. The UK Biobank resource with deep phenotyping and genomic data. *Nature* 2018; **562**:203-209.

[13] Karczewski KJ, Gupta R, Kanai M *et al*. Pan-UK Biobank GWAS improves discovery, analysis of genetic architecture, and resolution into ancestry-enriched effects. 2024. Doi: 10.1101/2024.03.13.24303864.

[14] Zhou W, Nielsen JB, Fritsche LG *et al.* Efficiently controlling for case-control imbalance and sample relatedness in large-scale genetic association studies. *Nat Genet* 2018; **50**:1335-1341.

[15] **Neale Lab** [<http://www.nealelab.is/uk-biobank/> (2 January 2025, date last accessed)]

**
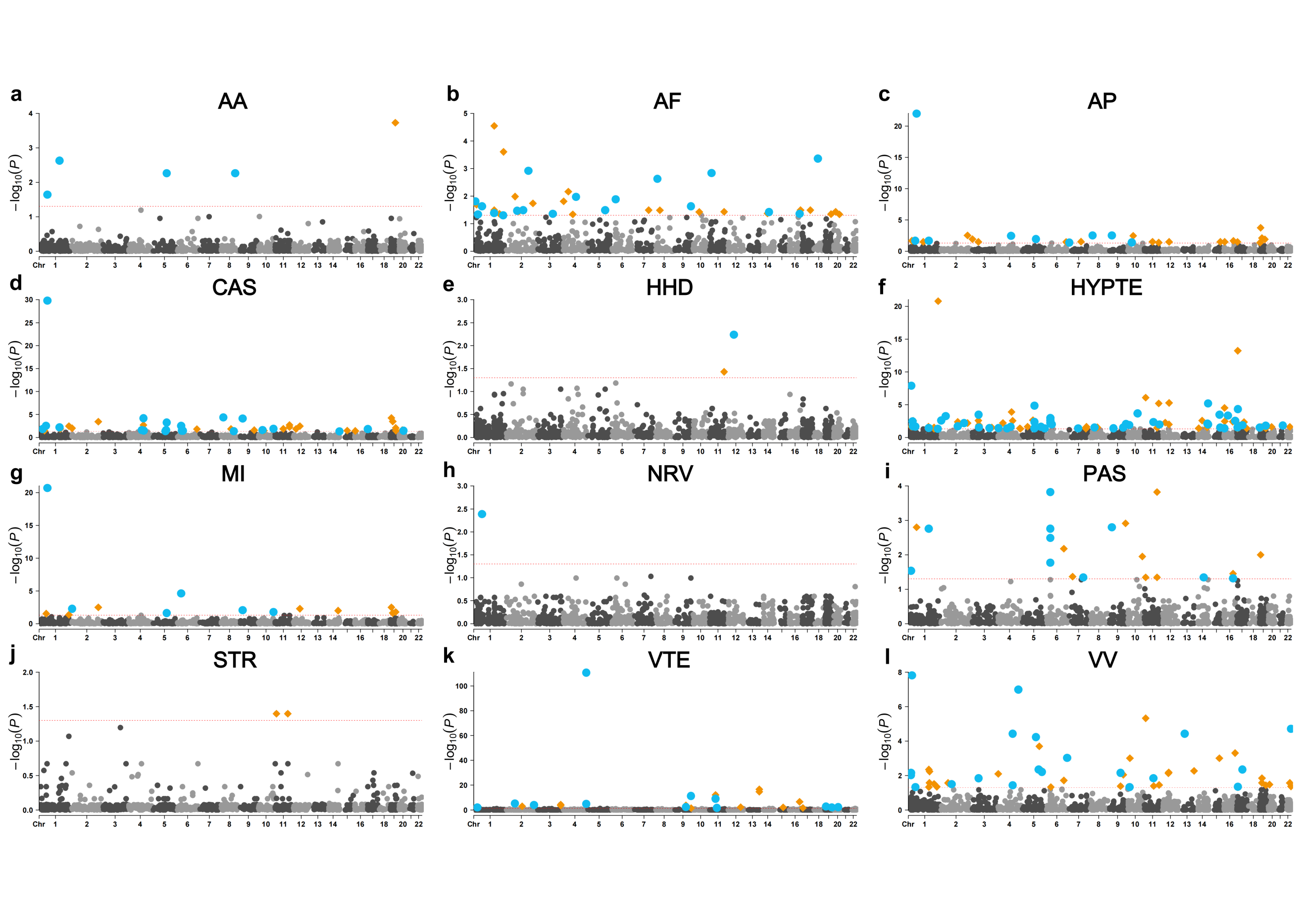
**

### **Supplementary Figure S1** Manhattan plots of protein-CVD associations identified by PWAS and TWAS. (a−l) Manhattan plots of protein-CVD associations identified by PWAS and TWAS for AA, AF, AP, CAS, HHD, HYPTE, MI, NRV, PAS, STR, VTE, and VV, respectively. Manhattan plots shows the chromosome against the –log_10_(*P* value) of PWAS results with each dot denoting a protein-CVD association. The red horizontal line denotes significant threshold of FDR corrected *P* < 0.05 in PWAS and *P* < 0.05 in TWAS, diamond denotes the PWAS-significant association, and circle denotes the significant association in both PWAS and TWAS. AA, aortic aneurysm; AF, atrial fibrillation and flutter; AP, angina pectoris; CAS, coronary atherosclerosis; HHD, hypertensive heart disease; HYPTE, hypertension; MI, myocardial infarction; NRV, rheumatic valve diseases; PAS, atherosclerosis, excluding cerebral, coronary and PAD; STR, stroke; VTE, venous thromboembolism; VV, varicose veins.


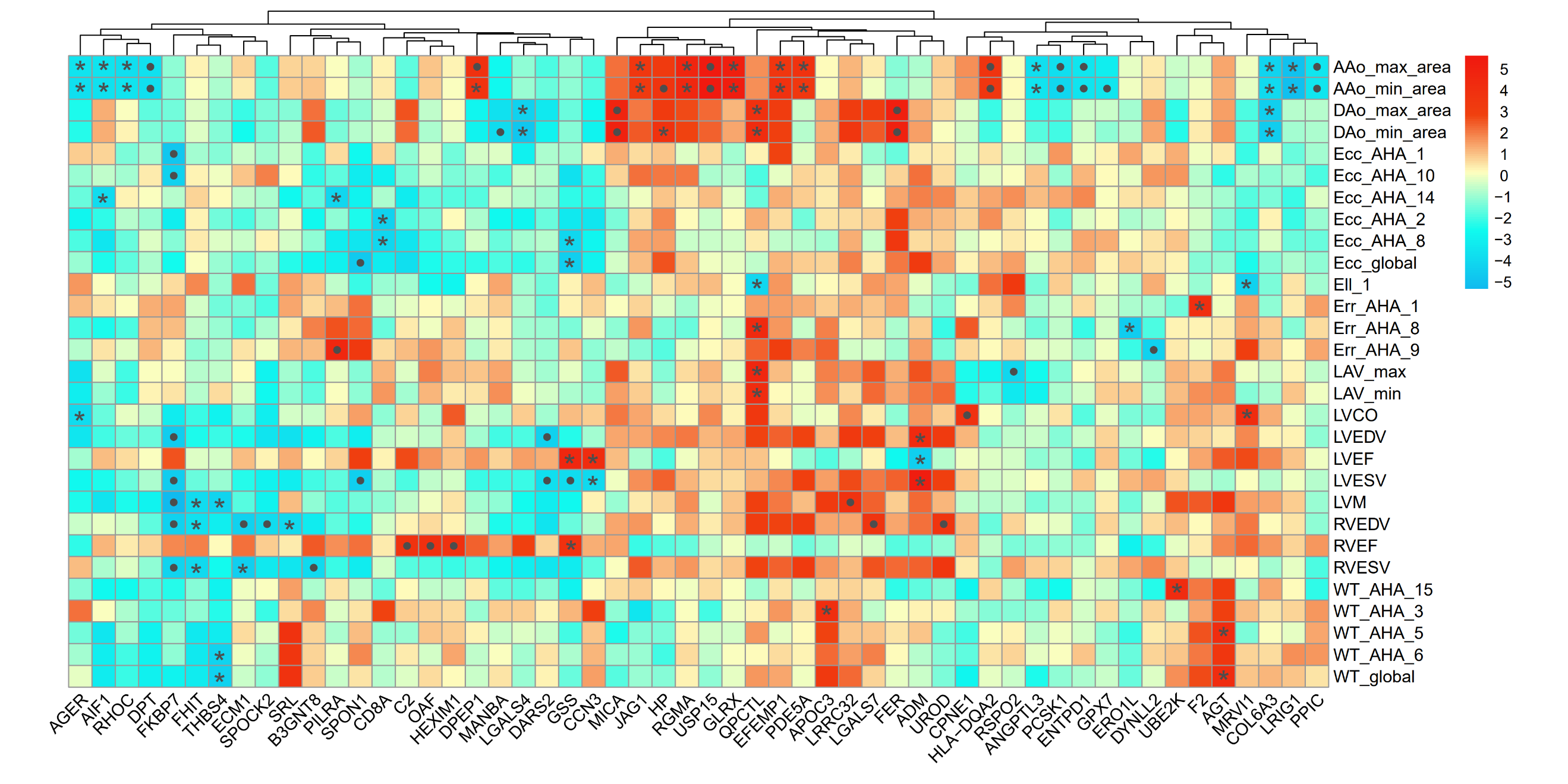


### **Supplementary Figure S2** Heatmap plot of associations between protein-coding genes and CMR traits identified by PWAS and TWAS. Cells are colored by PWAS Z-score. The rows denote CMR traits and the columns indicates genes. ^*^denotes the gene significantly associated with CMR trait in PWAS analysis. • denotes significant associations in both PWAS and TWAS analysis.


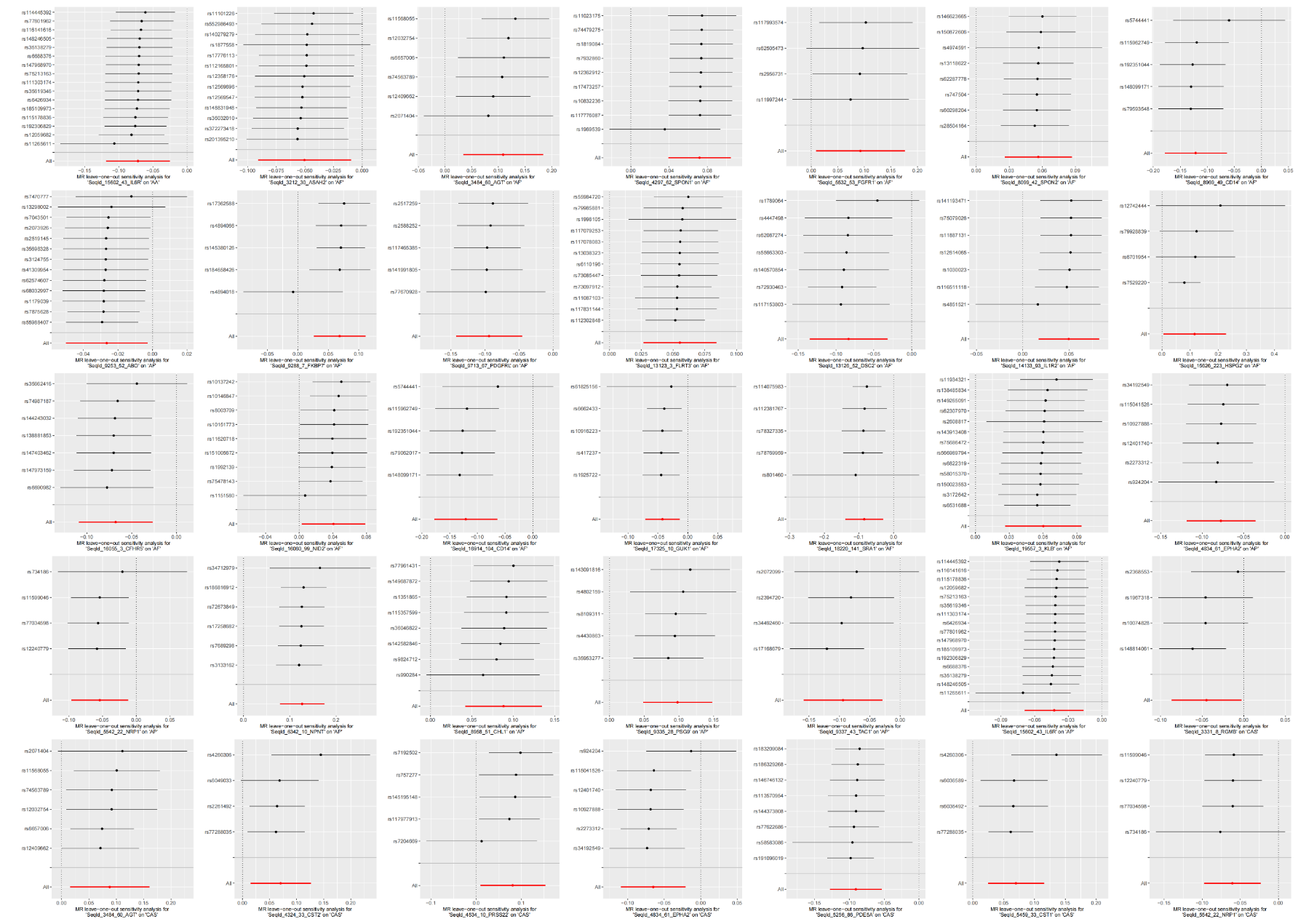


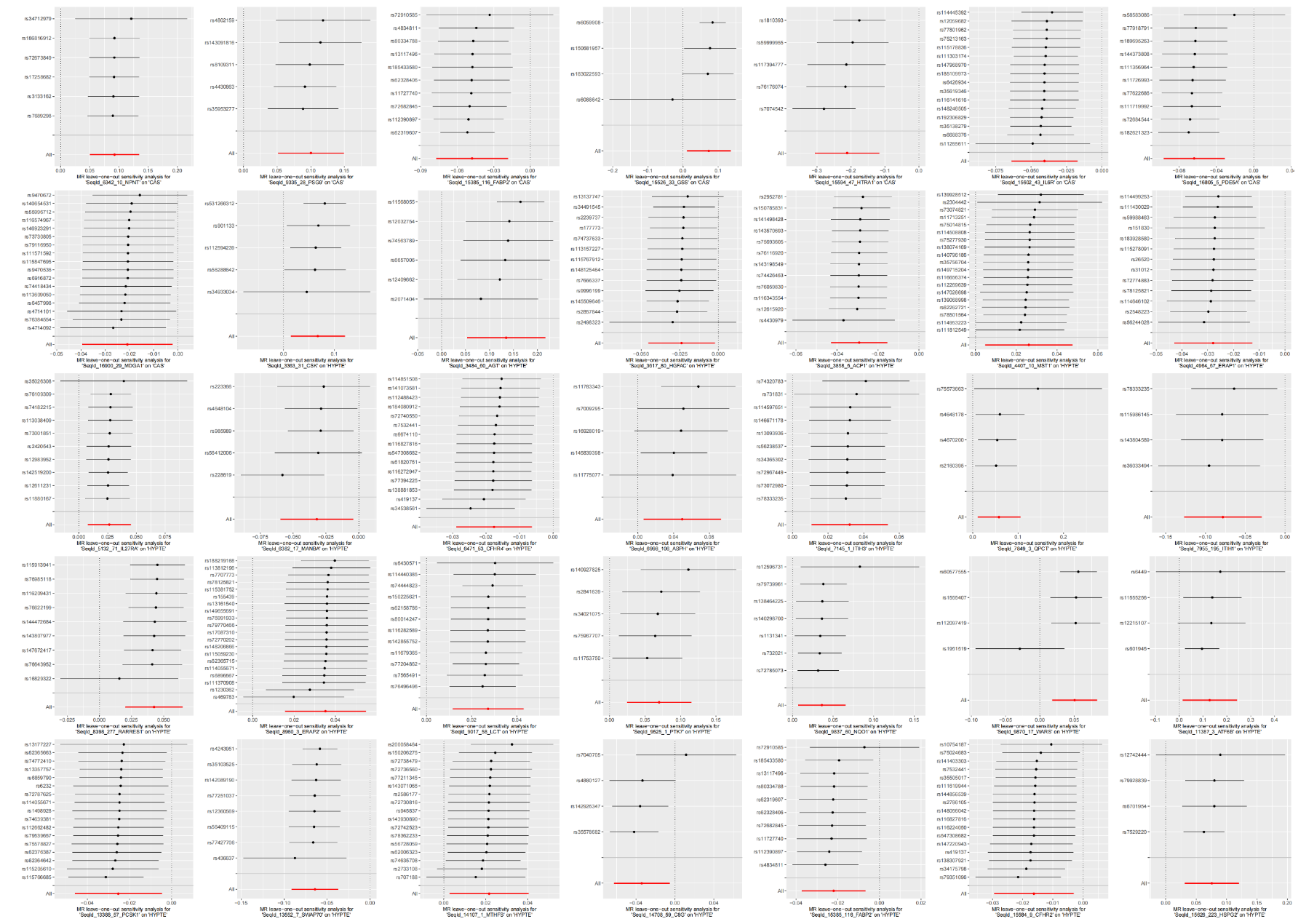


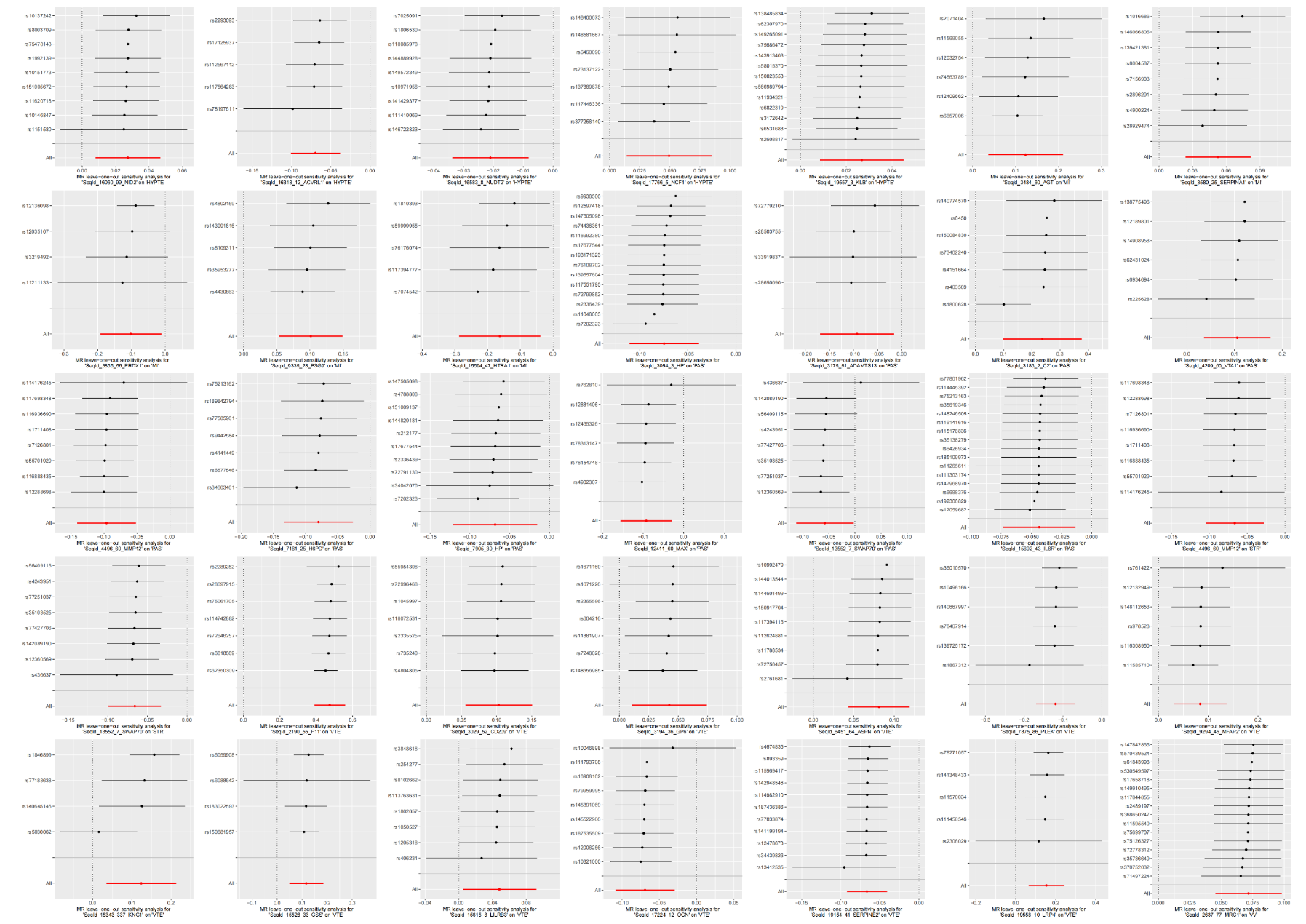


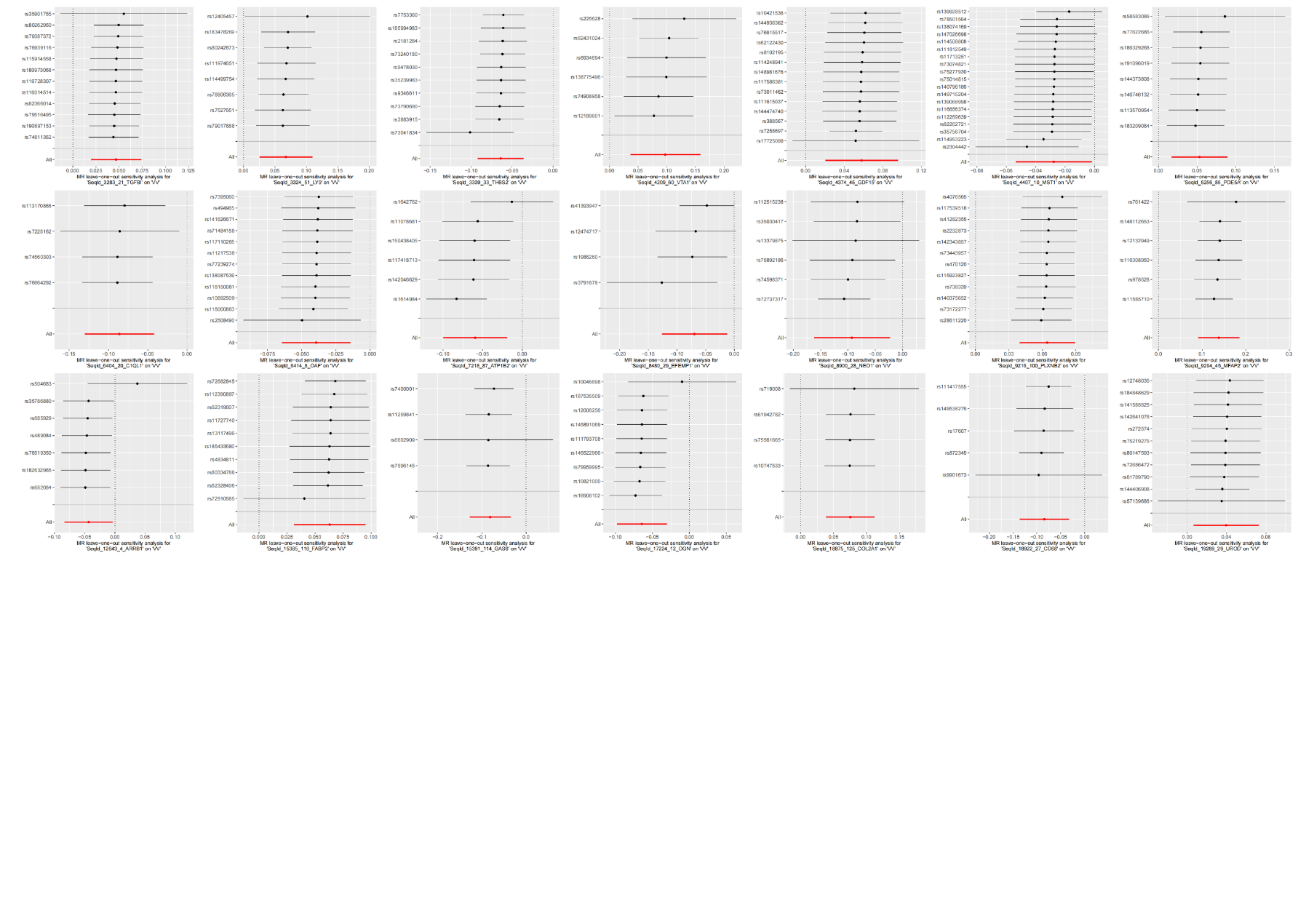


### **Supplementary Figure** S3 Leave-one-out plots for protein-CVD pairs in primary MR analysis. Leave-one-out analyses were only performed on 126 protein-CVD pairs with more than three instrumental variants. In each plot, the black line indicates the MR estimate (beta and 95% confidence interval) after removing one instrumental variant, and the red one represents the primary MR estimate (beta and 95% confidence interval).


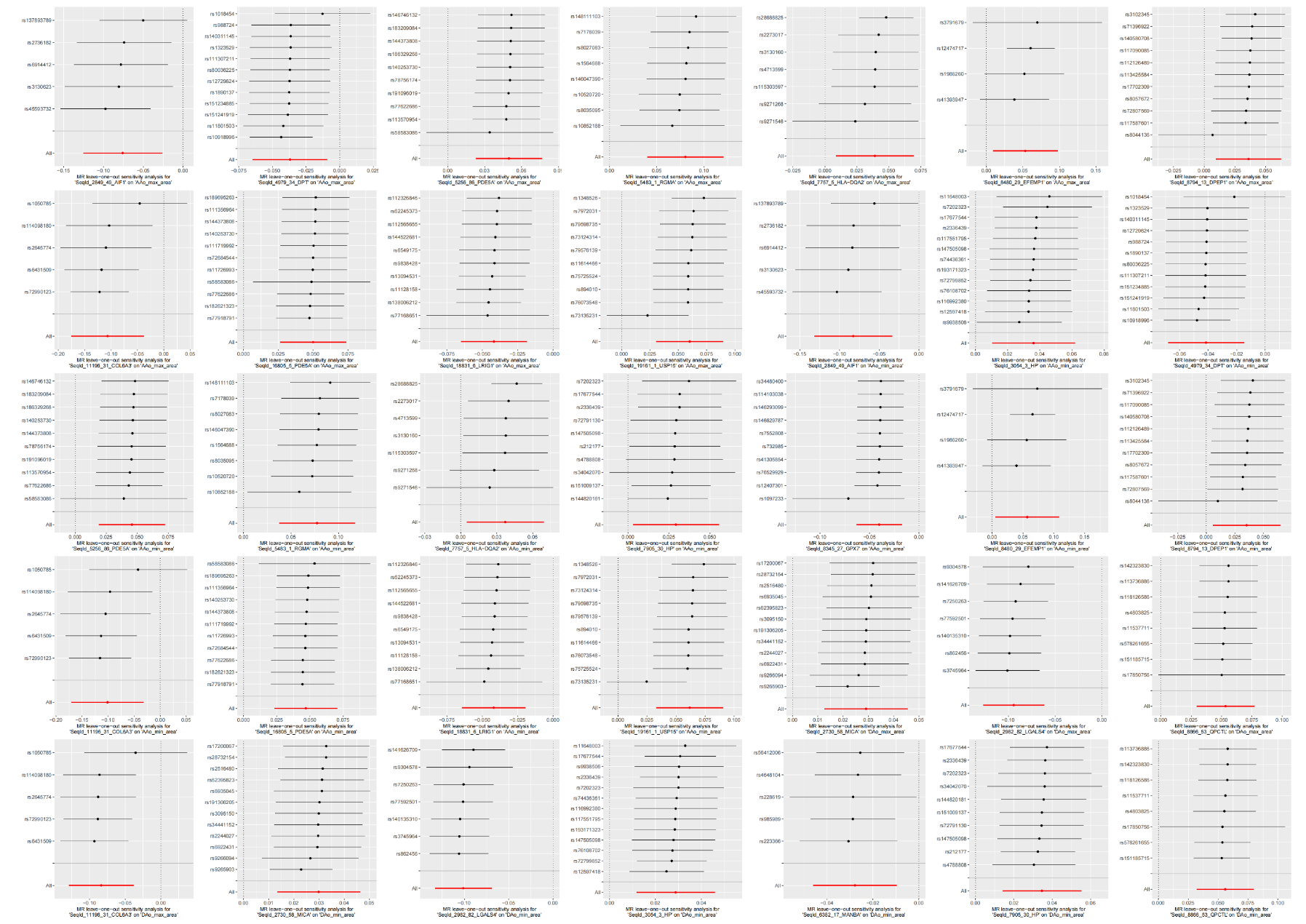


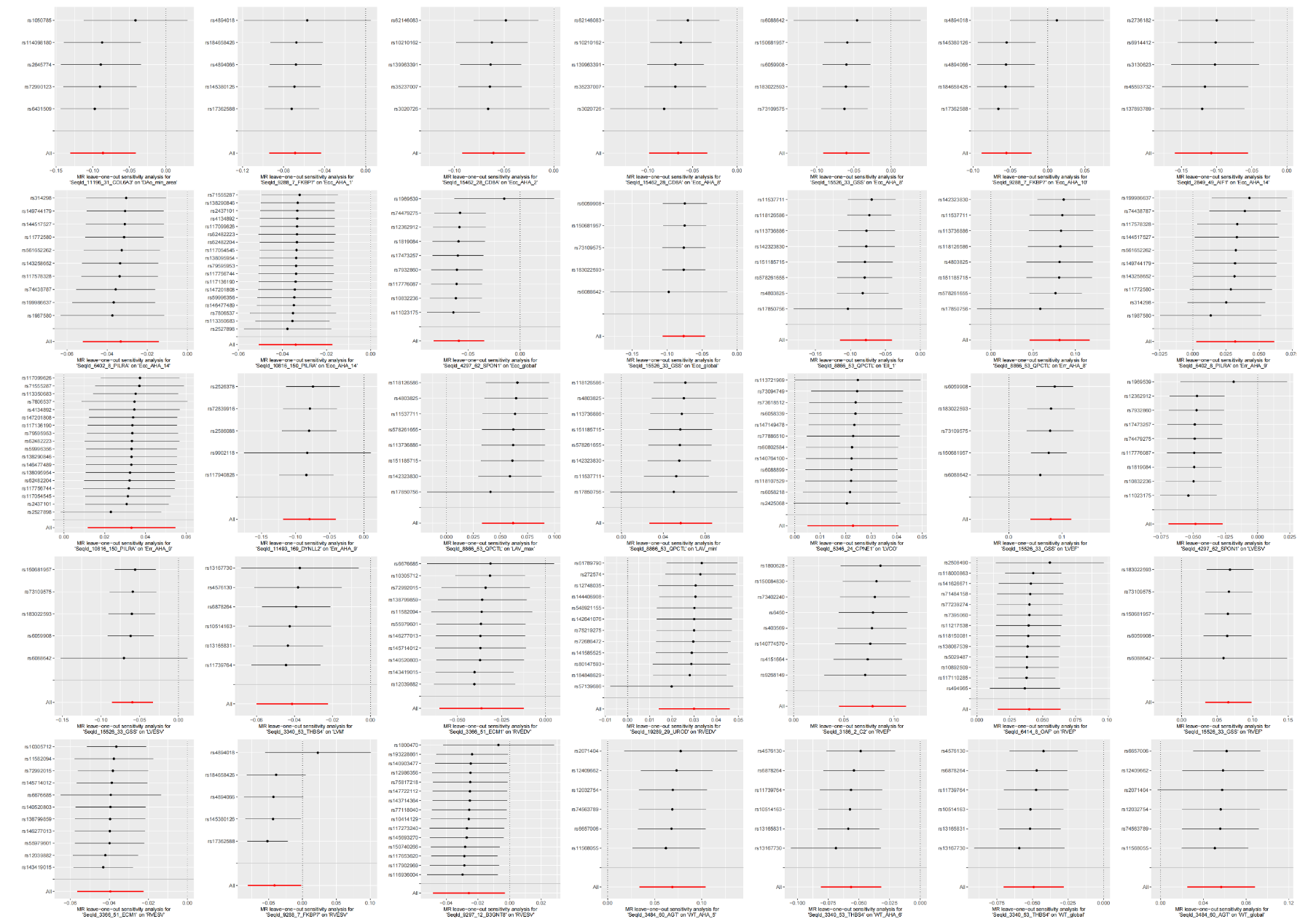


Supplementary Figure **S4** Leave-one-out plots for protein-CMR pairs in primary MR analysis. Leave-one-out analyses were only performed on 70 protein-CMR pairs with more than three instrumental variants. In each plot, the black line indicates the MR estimate (beta and 95% confidence interval) after removing one instrumental variant, and the red one represents the primary MR estimate (beta and 95% confidence interval).


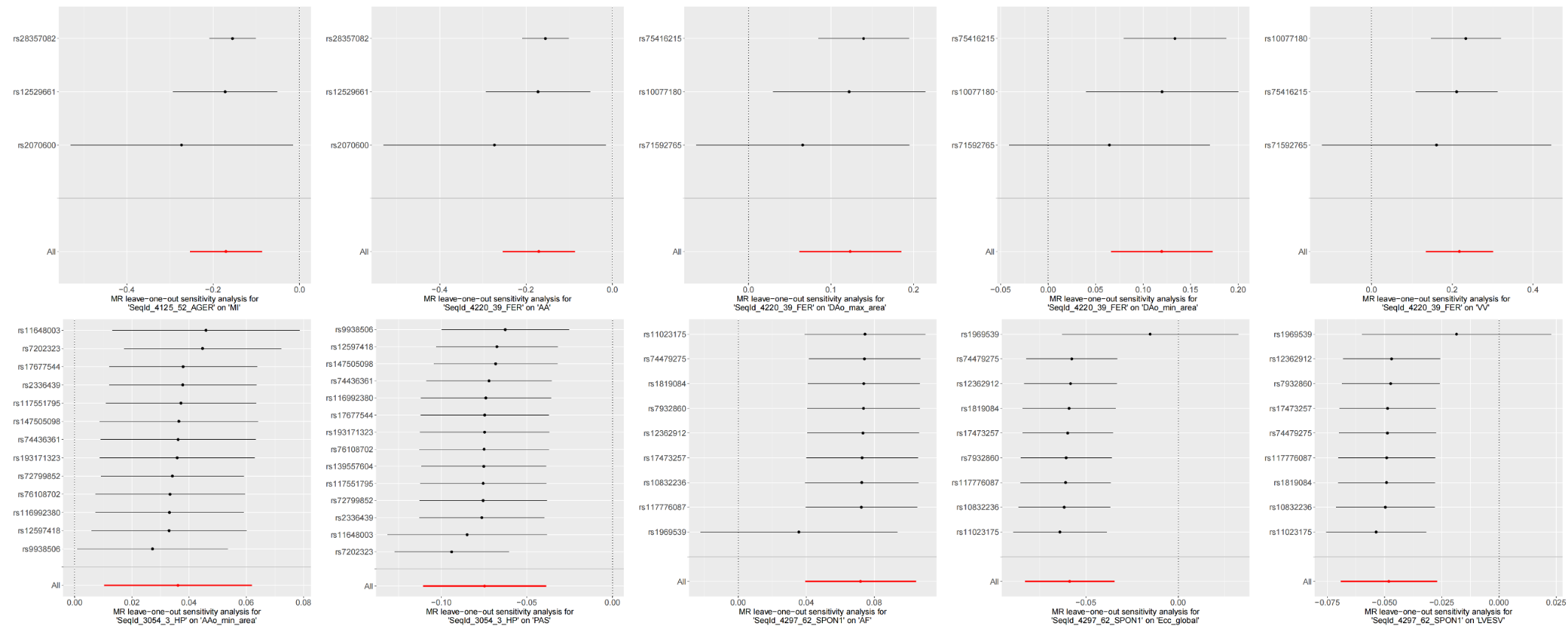


Supplementary Figure **S5** Leave-one-out plots for common proteins-associated protein-CVD and protein-CMR pairs in primary MR analysis. In each plot, the black line indicates the MR estimate (beta and 95% confidence interval) after removing one instrumental variant, and the red one represents the primary MR estimate (beta and 95% confidence interval).


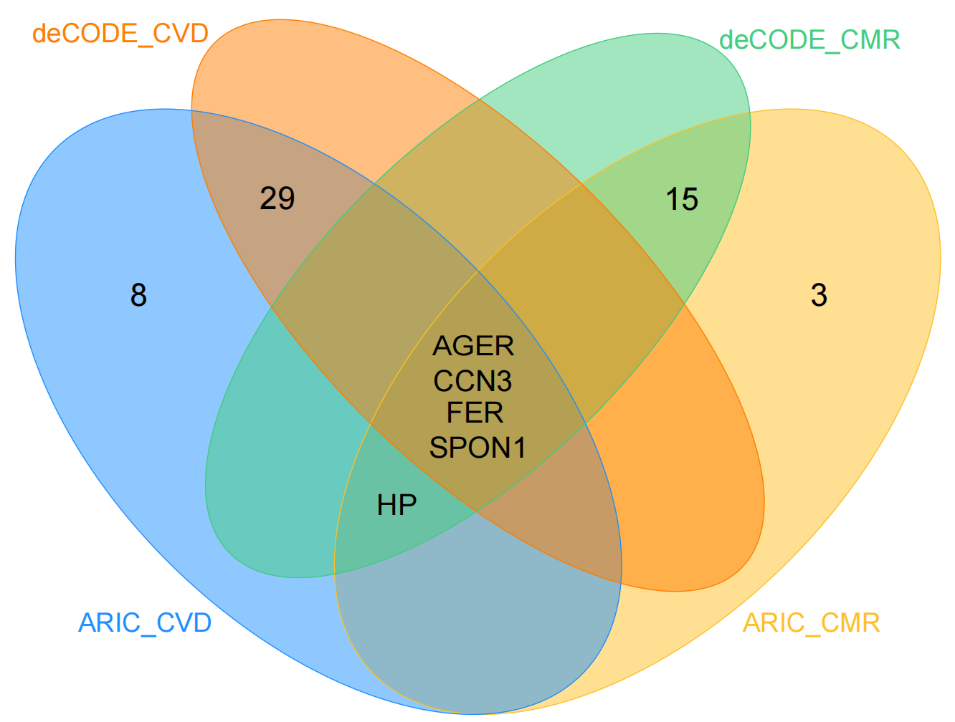


Supplementary Figure **S6** Venn plot of protein-coding genes identified using pQTL data from ARIC and deCODE. ARIC plasma pQTL data was used for discovery analysis and deCODE plasma pQTL data was used for replication analysis. ARIC, Atherosclerosis Risk in Communities; CVD, cardiovascular disease; CMR, cardiovascular magnetic resonance.


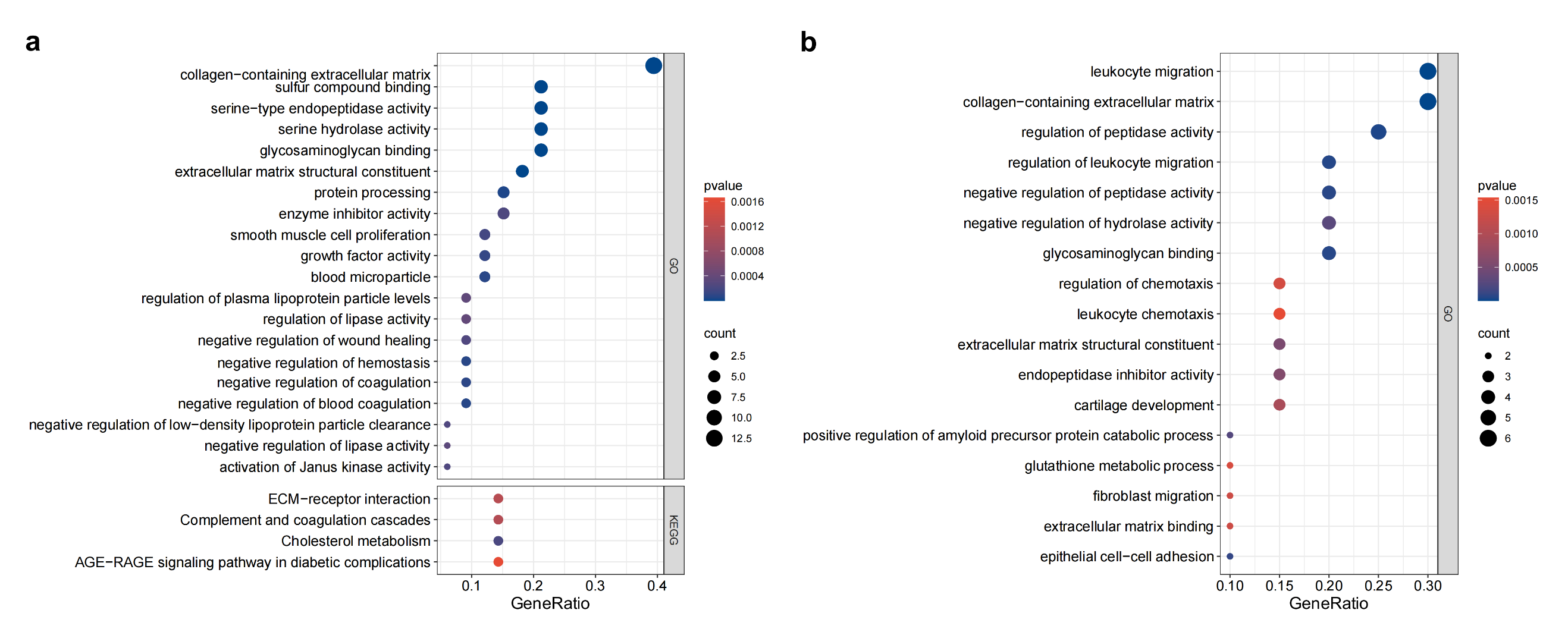


### **Supplementary Figure** S7 Significantly enriched biological pathways of protein-coding genes passed external replication. (a) GO and KEGG pathways of protein-coding genes associated with CVDs. (b) GO terms of protein-coding genes associated with CMR traits.

**
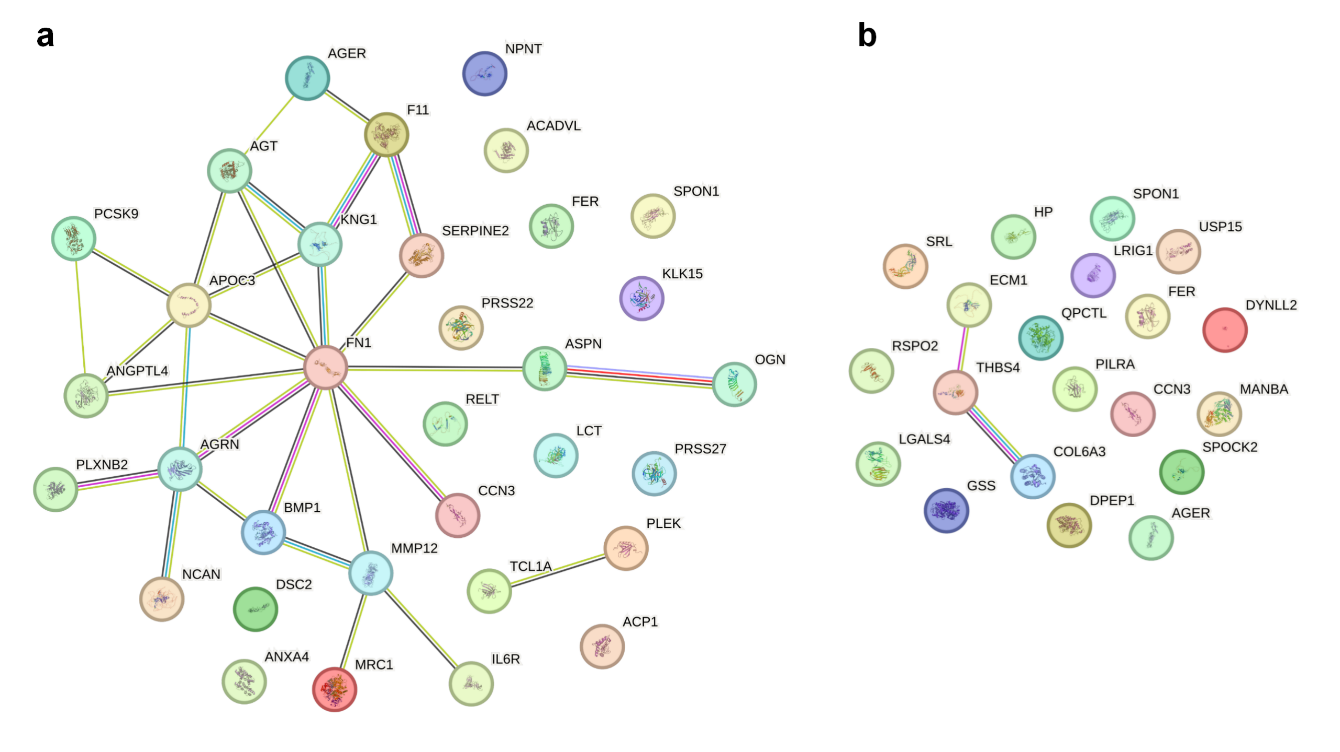
**

### **Supplementary Figure** S8 Protein-protein interaction (PPI) network of proteins passed external replication. (a) PPI network of CVD-associated proteins. (b) PPI network of CMR-associated proteins. Lines represent interactions between proteins. Blue line indicates known interaction from curated databases. Fuchsia line indicates known interaction that is experimentally determined. Green line indicates gene neighborhood and predicted interaction. Red line indicates gene fusion and predicted interaction. Dark blue line indicates gene co-occurrence and predicted interaction. Lime green line indicates textmining. Black line indicates co-expression. Lavender line indicates protein homology. Data information was from STRING database.

**
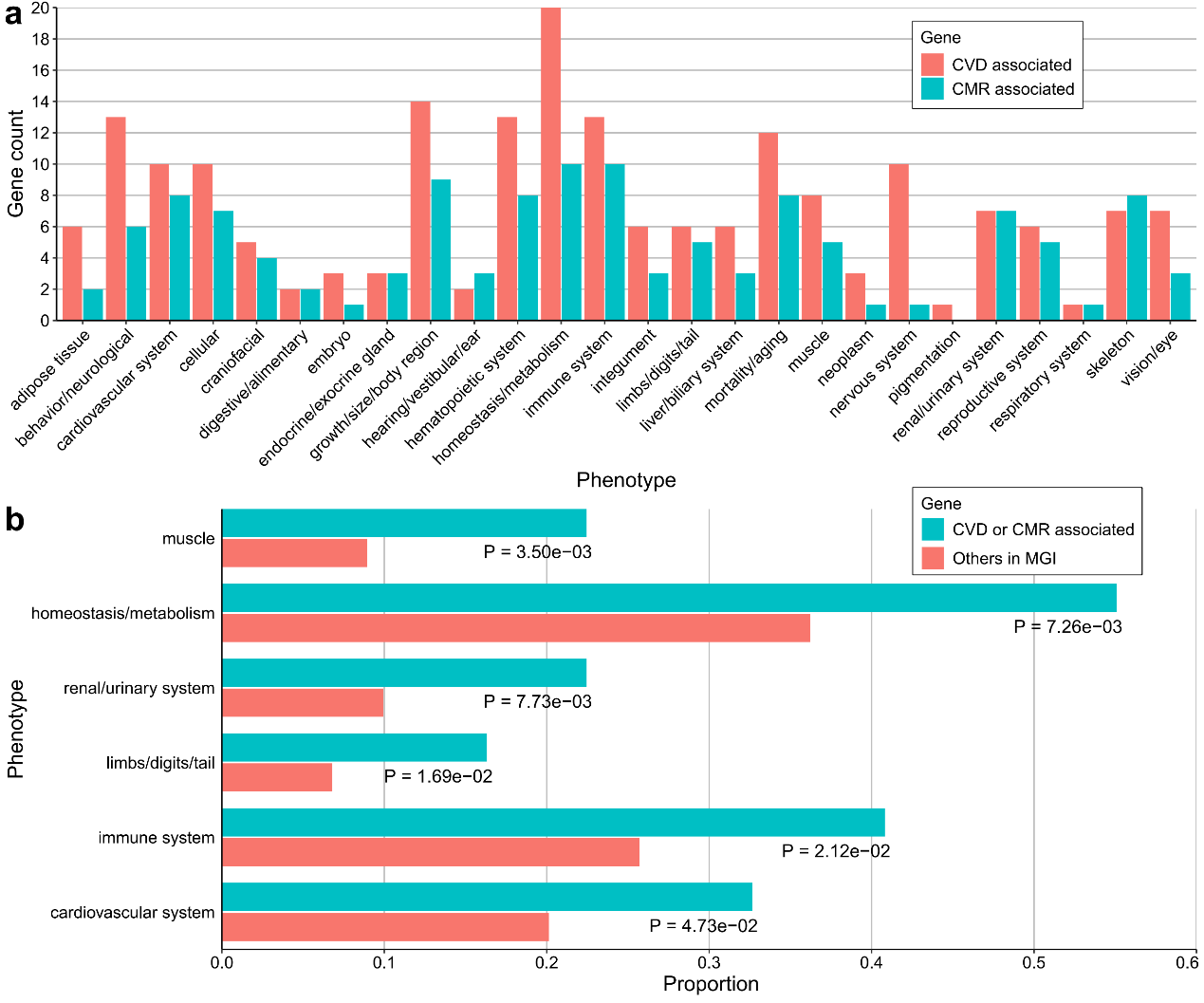
**

### **Supplementary Figure** S9 Phenotype enrichment analysis based on Mouse Genome Informatics platform. (a) Gene count of CVD-associated genes and CMR-associated genes classified to certain phenotype. (b) Phenotype enrichment analysis on protein-coding genes associated with CVDs or CMR traits.

**
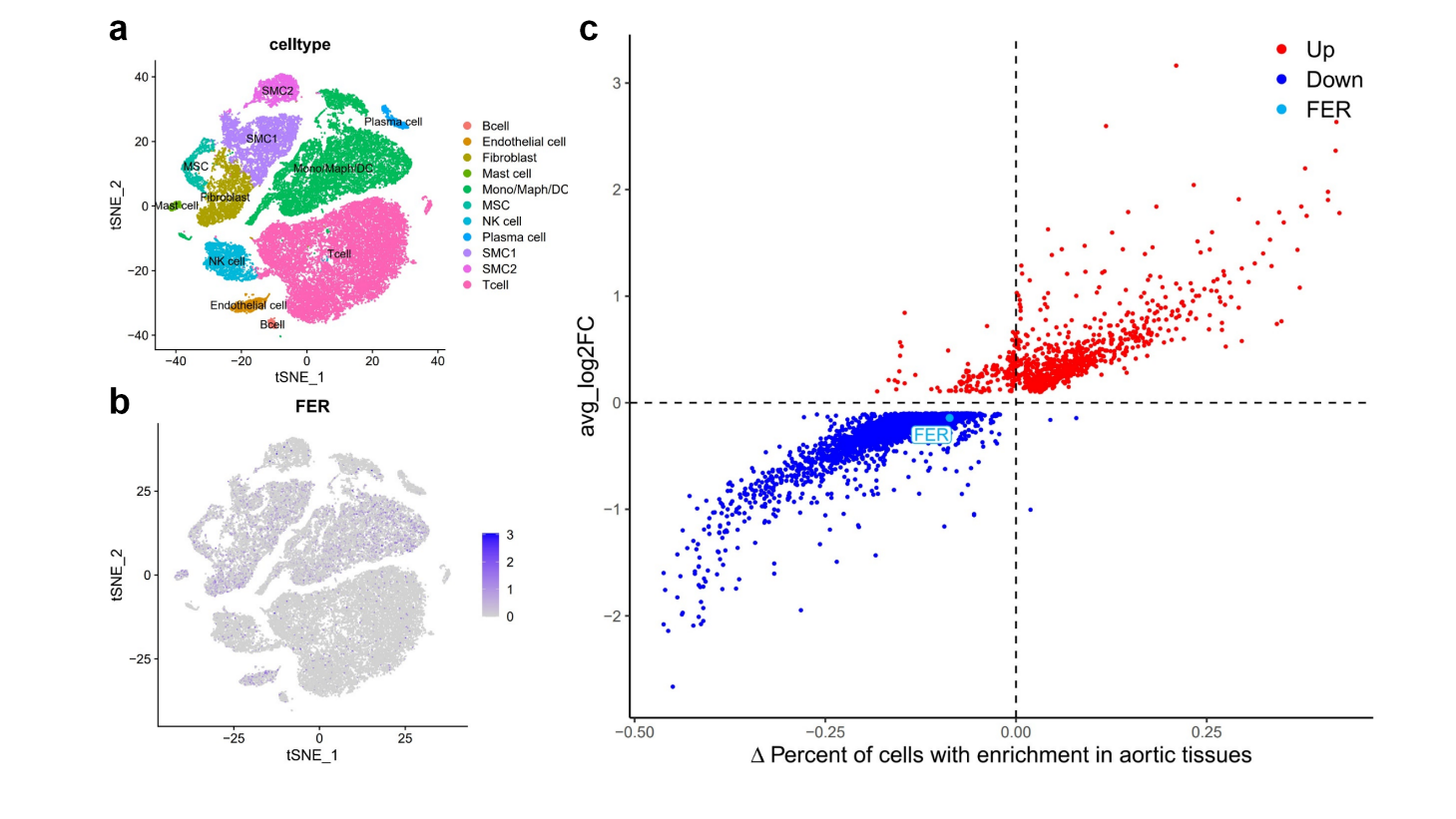
**

Supplementary Figure **S10** Single-cell expression of *FER* gene in 11 aortic tissue samples. (a) A total of 11 cell types identified. (b) Expression of *FER* in each cell type. (c) Volcano plot for differentially expressed genes between case group and control group at average log_2_FC > 0.1 and Bonferroni-corrected *P* < 0.01 level.


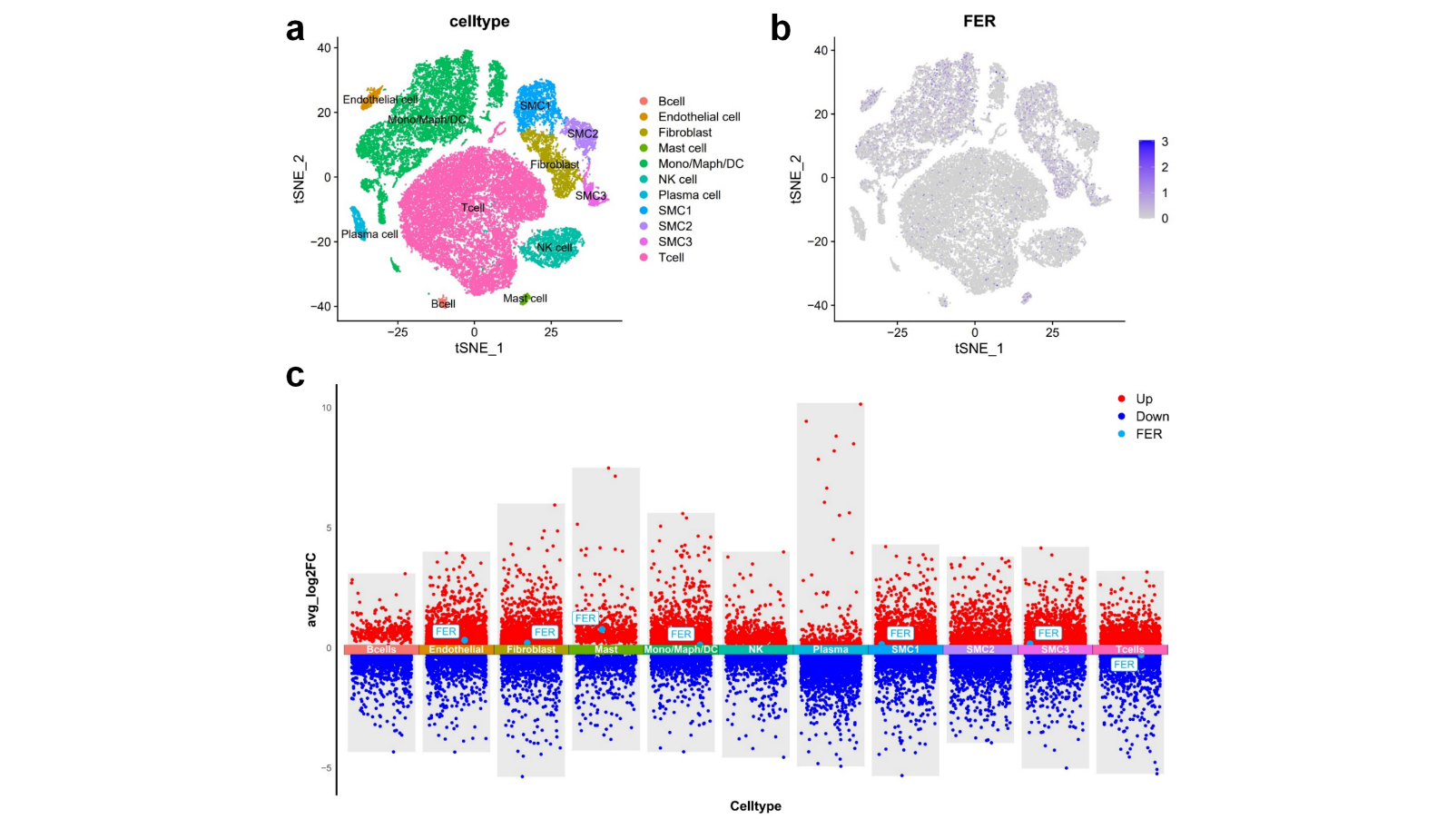


Supplementary Figure **S11** Single-cell expression of *FER* gene in eight aortic tissue samples with ascending thoracic aortic aneurysm. (a) A total of 11 cell types identified. (b) Expression of *FER* in each cell type. (c) Volcano plot for differentially expressed cell types of *FER* gene in case group at average log_2_FC > 0.1 and Bonferroni-corrected *P* < 0.01 level.


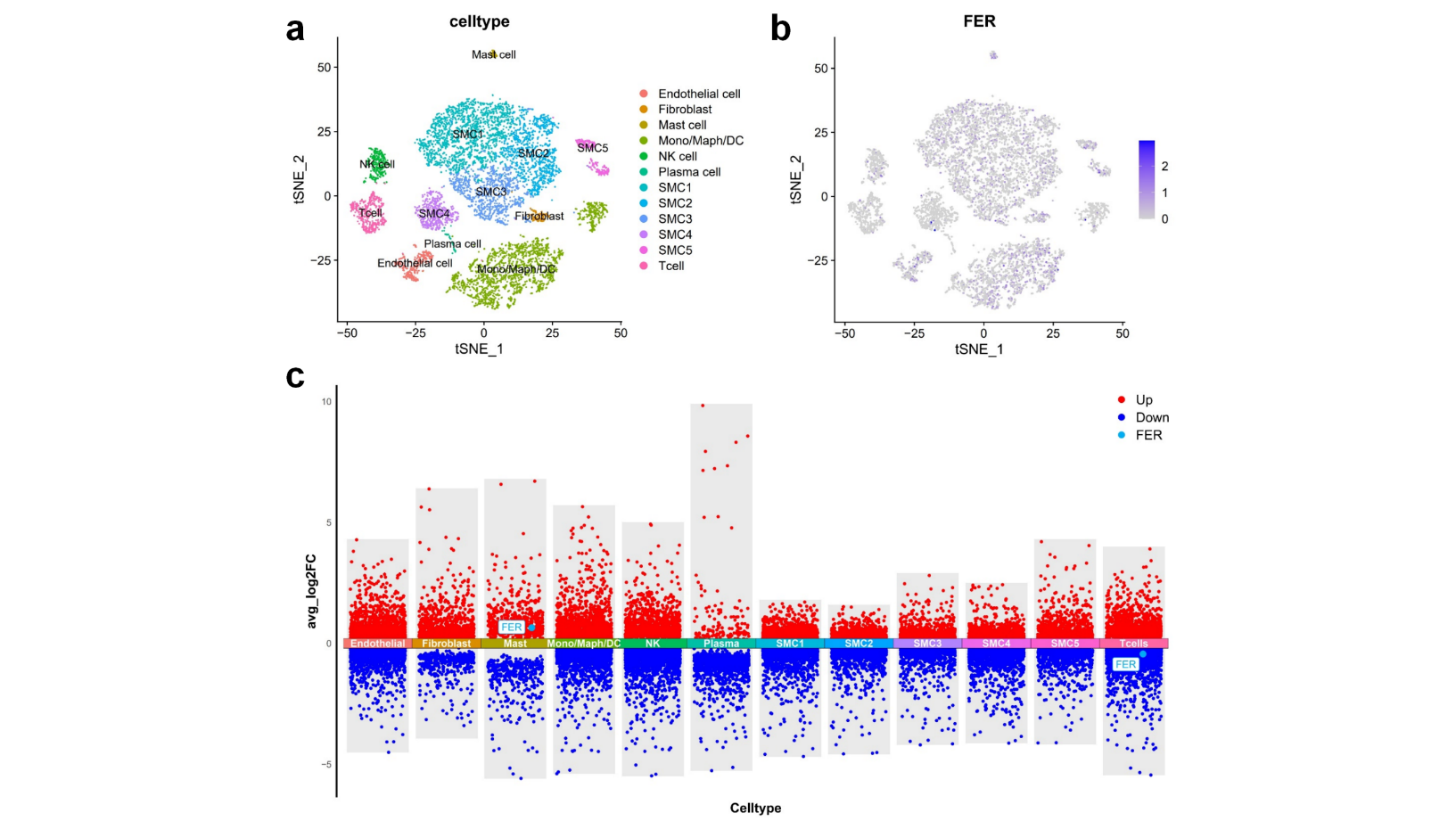


Supplementary Figure **S12** Single-cell expression of *FER* gene in three aortic tissue samples without aortic aneurysm. (a) A total of 12 cell types identified. (b) Expression of *FER* in each cell type. (c) Volcano plot for differentially expressed cell types of *FER* gene in control group at average log_2_FC > 0.1 and Bonferroni-corrected P < 0.01 level.
